# The crystal structure of a spherical vanadium complex encapsulating a nitrate anion

**DOI:** 10.1107/S205698902500739X

**Published:** 2025-08-27

**Authors:** Rian Jordaan, Christo van Staden, Dumisani V. Kama, Walter Purcell

**Affiliations:** a University of the Free State, Chemistry Department, Bloemfontein, South Africa; Universidade Federal do ABC, Brazil

**Keywords:** crystal structure, vanadium, caged nitrate ion

## Abstract

The crystal structure of a nitrate anion caged in spherical vanadium and oxygen structure surrounded by sodium hy­droxy and water solvent mol­ecules, systematic name poly[[hepta­deca­aqua­tetra­deca­oxidonona­sodium][penta­cosa­aqua­nitratoundeca­oxido­penta­deca­vanadium]], H_61_NNa_9_O_71_V_15_ is reported. The complex crystallizes in the non-centrosymmetric *Cc* space group and exhibits many inter- and intra­molecular hydrogen-bonding inter­actions. The complex contains V^IV^ and V^V^ centres, which are six-coordinate or octa­hedrally coordinated. The sodium atoms in this structure sit outside of the sphere with varying geometries and coordination numbers ranging from 5–8. The inter­actions between the sodium hy­droxy sheet and spherical vanadium contributes to the packing of the mol­ecules within the structure.

## Abstract 2

The crystal structure of a nitrate anion caged in spherical vanadium and oxygen structure surrounded by sodium hy­droxy and water solvent mol­ecules is reported.

## 1. Chemical context

Vanadium occurs naturally in the atmosphere, the earth’s crust and water reservoirs (Rehder, 2015 ▸). Vanadium is relatively versatile in that it can be prepared in multiple colourful oxidation states. However, only three of these, namely vanadium(III), (IV) and (V) are found in the environment and in organic systems.

Vanadyl and vanadate are oxyanions of vanadium in the IV and V oxidation states, respectively. Their aqueous chemistry is pH sensitive and the presence of prospective ligands along with variations in pH can result in the formation of multiple complexes with different coordination geometries. Unless the solution is kept at an acidic pH or the ion is bound to a stabilizing ligand, air oxidation will result in the rapid regeneration of vanadate (Crans & Tracey, 1998 ▸). When the pH of the vanadyl containing acidic solution is raised to 7–7.5, various oligomeric and polymeric species form, some of which may precipitate due to their low solubility (Krakowiak *et al.*, 2012 ▸).

Polyoxometalates (POMs) are a versatile class of compounds that contain multiple metal atoms connected through oxygen atoms or oxo bridges. Polyoxovanadates (POVs) consist of vanadium centres connected via bridging oxygen atoms. These groups or clusters often adopt highly symmetrical and well-defined frameworks (Pope & Müller 1991 ▸; Müller *et al.*, 1998 ▸). In spherical POV structures, the vanadium atoms are arranged into symmetrical, ball-shaped frameworks. POV complexes are generally composed of basic structural polyhedra such as square-pyramidal [VO_5_]^5−^ and [VO_5_]^6−^, tetra­hedral [VO_4_]^3−^ and the predominant octa­hedral [VO_6_]^7−^ and [VO_6_]^8−^, which are linked via bridging oxygen atoms (Monakhov *et al.*, 2015 ▸). These linkages often form ‘cages’ that may encapsulate heteroanions or alkali metal ions. The spherical geometry is commonly stabilized by a combination of mixed-valent vanadium (V^4+^/V^5+^) centres, hydrogen bonding, and electrostatic inter­actions with counter-cations (Linnenberg *et al.*, 2017 ▸). The incorporation of alkali metal ions, particularly sodium, plays a critical role in stabilizing the negative charge of the polyoxovanadate structure through electrostatic inter­actions, coordination, and hydrogen bonding, contributing to the overall structural integrity and assembly of the complex (Pope & Müller, 2007 ▸).

By adjusting the pH of an acidic aqueous solution of vanadium(IV), a single crystal of a spherical, POV-like vanadium structure with the mol­ecular formula H_61_NNa_9_O_71_V_15_ was isolated and is reported herein.

## 2. Structural commentary

**Figure 1:**
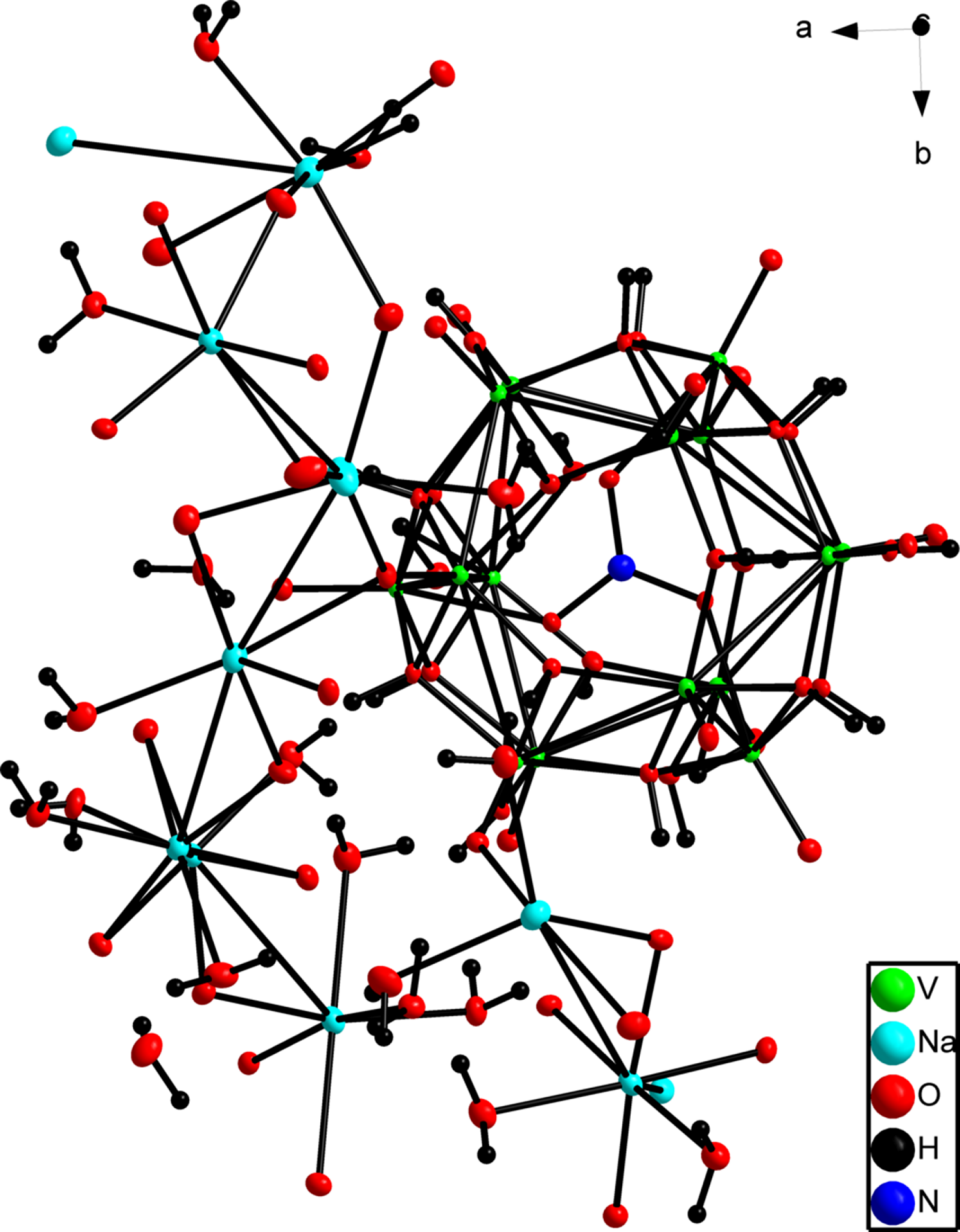
The asymmetric unit of the title compound. Displacement ellipsoids are shown at the 50% probability level.

The title compound crystallized in the non-centrosymmetric monoclinic space group, *Cc*. The polymeric vanadium hy­droxy structure has a nitrate anion in the centre of the spherical ball-shaped environment. Surrounding the latter are clustered sodium hydrate mol­ecules in an octa­hedral conformation. The 15 vanadium atoms within the sphere are made up of six-coordinate IV and V oxidation states with an octa­hedral geometry and some centres adopting a distorted octa­hedral geometry. The oxidation states were determined by bond-valence-sum (BVS) calculations. While it is common for most POV complexes to contain vanadium(V) only, with examples being deca­vanadates and Keggin-type POVS, spherical POV complexes, however, are usually comprised of V^IV^ and V^V^ centres, which is the case in this study. The V^IV^ centres in the structure can also be identified by short terminal bonds of approximately 1.58 Å (Schreiber *et al.*, 2022 ▸), which match the V—O bond lengths in this structure of 1.6 Å. These spherical POV structures are also stabilized by central heteroanions such as PO_4_^3−^ or NO_3_^−^, also seen in this structure. Furthermore in polyoxovanadate (POV) structures, alkali metal cations, particularly sodium (Na^+^), serve as charge-balancing counter-ions that neutralize the substantial negative charge of the POV anions (Amanchi & Das, 2018 ▸). The mol­ecular structure of this complex is illustrated in Fig. 1 ▸.

The *C*-centered space group contains a glide plane perpendicular to the *b* axis and along the *c*-axis by half of the lattice vector. Thus, for every atom observed at *x*, *y*, *z* an equivalent atom is present at *x*, −*y*, *z* + 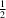. Furthermore, a twofold screw axis is observed parallel to the *b* axis. As a result of the non-centrosymmetric nature of the *Cc* space group, there is no inversion centre.

The nitrate is observed in the centre of a ball-like structure that is formed by the structural arrangement of vanadium pentoxide mol­ecules. The three semi-coordinated inter­molecular inter­actions between the oxygen atoms of the nitrate and vanadium atoms of the ball have an average distance of 2.287 (4). The vanadium pentoxide layer has an average V—V distances of 2.9384 (13).

Surrounding this vanadium ball is a clustered formation of sodium hydroxides and water mol­ecules. This sodium layer contributes to the overall stability of the structure through a variety of inter­molecular hydrogen bonds. The sodium atoms do not make up part of the sphere and these atoms have varying coordination numbers of 5 to 8 with different geometries (trigonal bypyrimidal and octa­hedral). The full list of potential hydrogen bonds is given in Table 1 ▸.

## 3. Supra­molecular features

**Figure 2:**
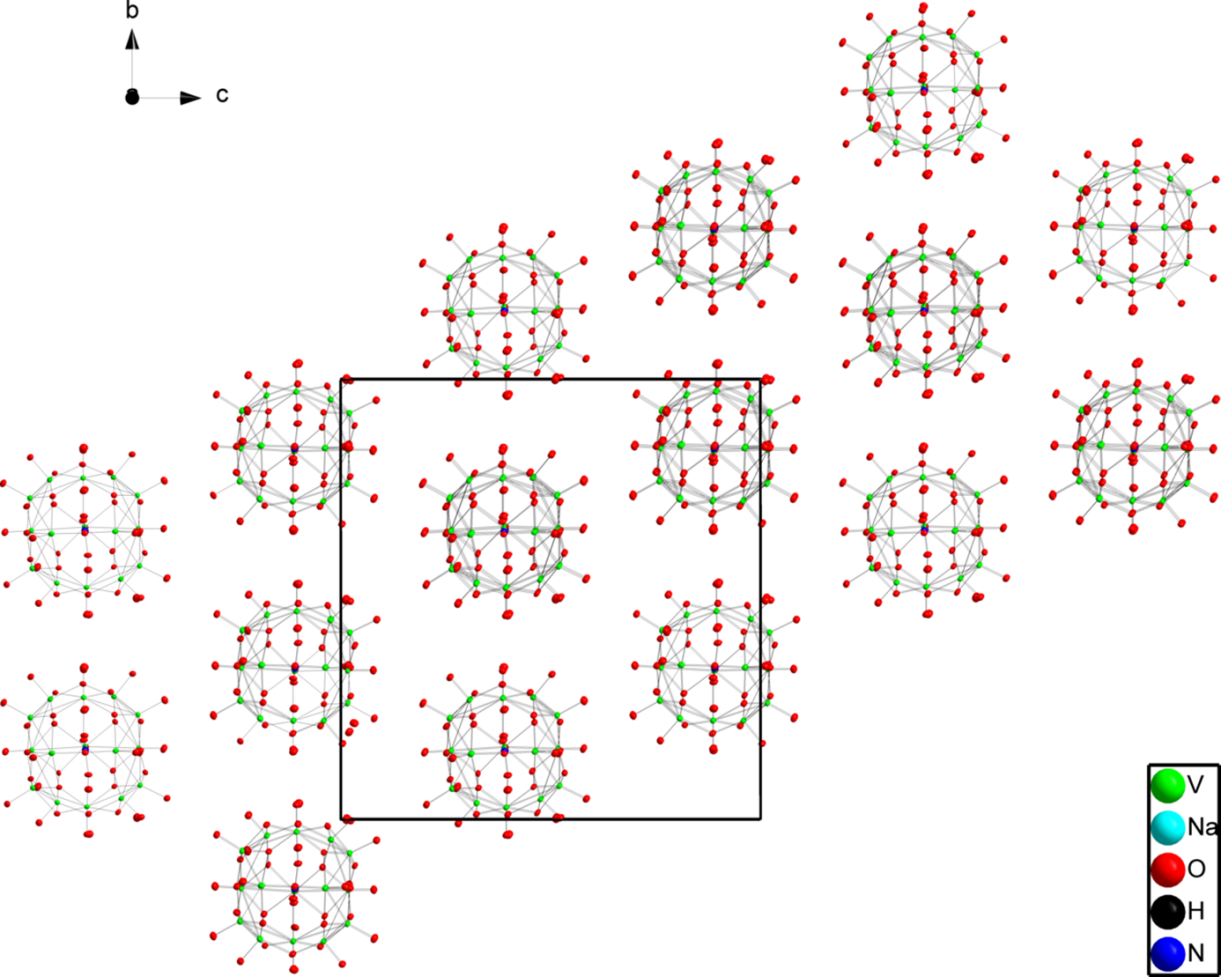
The packing of the spherical vanadium mol­ecules. All atoms and mol­ecules that are not directly part of the spherical structure was omitted for clarity. Displacement ellipsoids are drawn at the 50% probability level.

The components of the crystal structure discussed so far were based on the asymmetric unit. As mentioned earlier, the layered sodium cluster observed around the ball-like vanadium complex contributes to the stability of the structure that led to its crystallization. When the view of the structure is expanded to include other surrounding vanadium clusters, a diagonal packing pattern is observed along the *a*-axis direction as a result of the inter­molecular hydrogen bonds that form a chain of linked vanadium clusters as observed in Fig. 2 ▸.

## 4. Database survey

The novelty of the crystal structure was determined by doing a search on the Cambridge Structure Database (CSD; Groom *et al.*, 2016 ▸; accessed October 2024). First a unit-cell search was performed using *a* = 12.8252 Å, *b* = 22.629 Å, *c* = 21.717 Å and α = 90°, β = 96.279°, γ = 90° with a 10% tolerance on cell lengths and a 2° tolerance on the angles. This gave 14 hits, none of which contained vanadium, the closest cell parameters being for a gold complex. A formula search was performed for a ball-like vanadium structure with formula of V_15_O_39_N and no results were found. A similar spherical vanadium structure was observed in a protein crystallography study (Tito *et al.*, 2024 ▸; Ferraro *et al.*, 2023 ▸). These structures are similar with regard to the spherical nature, but differ in the number of vanadium atoms. Furthermore, the title structure crystallized with a sodium and oxygen sheet and water mol­ecules. To ensure a complete search was performed, a formula search was made using the various vanadium complexes used by Tito *et al.* (2024 ▸) and Ferraro *et al.* (2023 ▸) but no small mol­ecule crystal structures were found.

## 5. Synthesis and crystallization

All materials were used as received. Sodium metavanadate (1.0 g, ≥99.9%, Sigma Aldrich) was dissolved in 1.0 *M* sulfuric acid (95.0–98.0%, Sigma Aldrich), forming a yellow solution. The vanadate was reduced with excess sodium bis­ulfite (ACS Reagent, Sigma Aldrich) and stirred for 1 h whereupon the solution changed from yellow to blue. The pH of the solution was adjusted slowly with sodium hydroxide solutions (0.1 *M*, 1.0 *M*, 5.0 *M*, ≥98%, Sigma Aldrich), slowly effecting changes not exceeding 0.5 pH per addition. The pH adjustment was performed until the solution turned a transparent brown colour, the pH was then further adjusted and upon the formation of a brown precipitate, the solution was covered and left at room temperature for 24 h pending the formation of the brown crystals, which were isolated and analysed. The sulfuric acid (95.0–98.0%, Sigma Aldrich) used in this experiment played a critical role, as the nitro­gen atom present in the final structure is attributed to nitrate impurities inherent in the acid.

## 6. Refinement

Crystal data, data collection and structure refinement details are summarized in Table 2 ▸. As a result of the polymeric nature of this structure and the clustered water mol­ecules, not all hydrogen atoms were successfully located due to the low scattering factors. The hydrogen atoms were placed in geometrically idealized positions and refined as riding.

## Supplementary Material

Crystal structure: contains datablock(s) I. DOI: 10.1107/S205698902500739X/ee2018sup1.cif

Structure factors: contains datablock(s) I. DOI: 10.1107/S205698902500739X/ee2018Isup3.hkl

CCDC reference: 2364387

Additional supporting information:  crystallographic information; 3D view; checkCIF report

## Figures and Tables

**Table 1:** Hydrogen-bond geometry (Å, °)

*D*—H⋯*A*	*D*—H	H⋯*A*	*D*⋯*A*	*D*—H⋯*A*
O0*P*—H0*P*⋯O02*G*^i^	1.00	1.76	2.752 (5)	174
O36—H36⋯O025^i^	1.00	1.80	2.803 (5)	177
O17—H17⋯O56	1.00	1.74	2.726 (5)	169
O13—H13⋯O54^ii^	1.00	1.74	2.715 (6)	165
O5—H5⋯O55^i^	1.00	1.87	2.791 (5)	152
O9—H9⋯O49	1.00	1.80	2.729 (6)	153
O19—H19⋯O01*E*	1.00	1.82	2.789 (5)	163
O27—H27⋯O023^iii^	0.87 (1)	2.07 (5)	2.790 (6)	139 (6)
O10—H10⋯O01*U*	1.00	1.86	2.857 (6)	174
O12—H12⋯O50^ii^	1.00	1.73	2.718 (5)	169
O7—H7⋯O59^iv^	1.00	1.76	2.751 (6)	173
O56—H56*A*⋯O17	0.87	1.88	2.726 (5)	165
O56—H56*B*⋯O57	0.87	1.97	2.753 (6)	148
O50—H50*A*⋯O12^v^	0.87	1.91	2.718 (5)	154
O50—H50*B*⋯O47	0.87	1.91	2.766 (6)	168
O48—H48*A*⋯O33^v^	0.87	1.85	2.719 (6)	174
O58—H58*B*⋯O35	0.87	2.01	2.748 (6)	142
O54—H54*A*⋯O13^v^	0.87	2.01	2.715 (6)	137
O54—H54*B*⋯O38^vi^	0.87	1.93	2.745 (6)	155
O40—H40*A*⋯O50^i^	0.87	1.89	2.727 (6)	160
O40—H40*B*⋯O01*E*^iii^	0.87	1.93	2.752 (6)	156
O01*U*—H01*A*⋯O10	0.87	2.04	2.857 (6)	157
O01*W*—H01*C*⋯O02*G*	0.87	2.06	2.795 (6)	142
O01*W*—H01*D*⋯O31^vii^	0.87	2.05	2.883 (6)	161
O44—H44*B*⋯O58^viii^	0.88	1.88	2.740 (6)	168
O60—H60*A*⋯O46^ix^	0.87	1.92	2.766 (6)	164
O60—H60*B*⋯O33^vi^	0.87	2.00	2.860 (6)	167
O022—H02*A*⋯O48^x^	0.88	1.98	2.795 (6)	154
O022—H02*B*⋯O02*J*	0.87	2.09	2.895 (6)	152
O47—H47*A*⋯O50	0.88	2.05	2.766 (6)	139
O47—H47*B*⋯O57^viii^	0.88	2.14	2.864 (6)	139
O025—H02*C*⋯O60^xi^	0.87	1.90	2.763 (6)	169
O025—H02*D*⋯O02*K*	0.87	1.95	2.791 (7)	162
O41—H41*A*⋯O11^iii^	0.87	1.91	2.746 (6)	159
O41—H41*B*⋯O56^xii^	0.87	1.92	2.760 (6)	162
O02*G*—H02*E*⋯O01*W*	0.87	2.05	2.795 (6)	142
O02*G*—H02*F*⋯O02*K*	0.87	2.02	2.828 (7)	154
O38—H38*A*⋯O54^xiii^	0.87	1.88	2.745 (6)	175
O38—H38*B*⋯O55^xiii^	0.87	2.22	2.832 (6)	127
O02*K*—H02*G*⋯O26^viii^	0.88	2.21	2.769 (6)	121
O57—H57*A*⋯O56	0.87	1.89	2.753 (6)	171
O57—H57*B*⋯O47^ix^	0.87	2.10	2.864 (6)	146

Symmetry codes: (i) 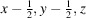; (ii) 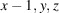; (iii) 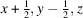; (iv) 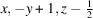; (v) 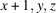; (vi) 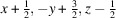; (vii) 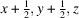; (viii) 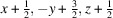; (ix) 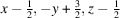; (x) 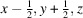; (xi) 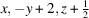; (xii) 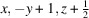; (xiii) 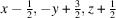.

**Table 2:** Experimental details

Crystal data	
Chemical formula	H_61_NNa_9_O_71_V_15_
*M*_r_	2182.50
Crystal system, space group	Monoclinic, *Cc*
Temperature (K)	100
*a*, *b*, *c* (Å)	12.8252 (11), 22.629 (3), 21.717 (2)
β (°)	96.279 (3)
*V* (Å^3^)	6264.8 (11)
*Z*	4
Radiation type	Mo *K*α
μ (mm^−1^)	2.33
Crystal size (mm)	0.16 × 0.14 × 0.03

Data collection	
Diffractometer	Bruker D8 Venture 4K Kappa Photon III C28
Absorption correction	Multi-scan (*SADABS*; Krause *et al.*, 2015 ▸)
*T*_min_, *T*_max_	0.632, 0.746
No. of measured, independent and observed [*I* > 2σ(*I*)] reflections	73216, 14656, 13827
*R*_int_	0.053
(sin θ/λ)_max_ (Å^−1^)	0.668

Refinement	
*R*[*F*^2^ > 2σ(*F*^2^)], *wR*(*F*^2^), *S*	0.032, 0.082, 1.07
No. of reflections	14656
No. of parameters	906
No. of restraints	23
H-atom treatment	H atoms treated by a mixture of independent and constrained refinement
Δρ_max_, Δρ_min_ (e Å^−3^)	0.80, −0.76
Absolute structure	Flack *x* determined using 6092 quotients [(*I*^+^)−(*I*^−^)]/[(*I*^+^)+(*I*^−^)] (Parsons *et al.*, 2013 ▸)
Absolute structure parameter	0.014 (6)

Computer programs: *APEX3* (Bruker, 2016 ▸), *SAINT* V8.40B (Bruker, 2016 ▸), *SHELXT2018/2* (Sheldrick, 2015*a* ▸), *SHELXL2019/3* (Sheldrick, 2015*b* ▸), *OLEX2* 1.5 (Dolomanov *et al.*, 2009 ▸), *DIAMOND* (Brandenburg & Putz, 2023 ▸), *publCIF* (Westrip, 2010 ▸).

## supplementary crystallographic information

### Poly[[heptadecaaquatetradecaoxidononasodium] [pentacosaaquanitratoundecaoxidopentadecavanadium]] Crystal data

**Table:**

[Na_9_O_14_(H_2_O)_17_][V_15_O_11_(OH)_25_(NO_3_)]·H_2_O	*F*(000) = 4320
*M**_r_* = 2182.50	*D*_x_ = 2.314 Mg m^−^^3^
Monoclinic, *C**c*	Mo *K*α radiation, λ = 0.71073 Å
*a* = 12.8252 (11) Å	Cell parameters from 9786 reflections
*b* = 22.629 (3) Å	θ = 2.5–28.3°
*c* = 21.717 (2) Å	µ = 2.33 mm^−^^1^
β = 96.279 (3)°	*T* = 100 K
*V* = 6264.8 (11) Å^3^	Plate, black
*Z* = 4	0.16 × 0.14 × 0.03 mm

### Poly[[heptadecaaquatetradecaoxidononasodium] [pentacosaaquanitratoundecaoxidopentadecavanadium]] Data collection

**Table:**

Bruker D8 Venture 4K Kappa Photon III C28 diffractometer	13827 reflections with *I* > 2σ(*I*)
ω and φ scans	*R*_int_ = 0.053
Absorption correction: multi-scan (SADABS; Krause *et al.*, 2015)	θ_max_ = 28.3°, θ_min_ = 1.8°
*T*_min_ = 0.632, *T*_max_ = 0.746	*h* = −17→17
73216 measured reflections	*k* = −30→30
14656 independent reflections	*l* = −28→28

### Poly[[heptadecaaquatetradecaoxidononasodium] [pentacosaaquanitratoundecaoxidopentadecavanadium]] Refinement

**Table:**

Refinement on *F*^2^	Hydrogen site location: mixed
Least-squares matrix: full	H atoms treated by a mixture of independent and constrained refinement
*R*[*F*^2^ > 2σ(*F*^2^)] = 0.032	*w* = 1/[σ^2^(*F*_o_^2^) + (0.0355*P*)^2^ + 20.6951*P*] where *P* = (*F*_o_^2^ + 2*F*_c_^2^)/3
*wR*(*F*^2^) = 0.082	(Δ/σ)_max_ = 0.001
*S* = 1.07	Δρ_max_ = 0.80 e Å^−^^3^
14656 reflections	Δρ_min_ = −0.76 e Å^−^^3^
906 parameters	Absolute structure: Flack *x* determined using 6092 quotients [(*I*^+^)-(*I*^-^)]/[(*I*^+^)+(*I*^-^)] (Parsons *et al.*, 2013)
23 restraints	Absolute structure parameter: 0.014 (6)
Primary atom site location: dual	

### Poly[[heptadecaaquatetradecaoxidononasodium] [pentacosaaquanitratoundecaoxidopentadecavanadium]] Special details

**Table:**

Experimental. The crystal structure reported herein was collected using a Bruker D8 Quest Eco Chi Photon II CPAD diffractometer with φ and ω-scans at 100?K. Graphite monochromated Mo Kα radiation with wavelength λ = 0.71073?Å was used. The cell refinement and data reduction were done with SAINT-Plus with XPREP included. Absorption corrections were obtained by using the multi-scan technique and the SADABS software package. (Bruker, 2016) (Bruker, 2005) (Bruker, 2012) The structure solution and refinement was performed using SHELXT and SHELXL-2013 in conjunction with the OLEX software suite. (Sheldrick, 2015) (Sheldrick, 2008) (Dolomanov *et al.* 2009) (Farrugia, 2012) The molecular structures were drawn using DIAMOND. (Brandenburg & Putz, 2023) All atoms except for hydrogen atoms were anisotropically refined and thermal ellipsoids drawn at 50% probability.
Geometry. All esds (except the esd in the dihedral angle between two l.s. planes) are estimated using the full covariance matrix. The cell esds are taken into account individually in the estimation of esds in distances, angles and torsion angles; correlations between esds in cell parameters are only used when they are defined by crystal symmetry. An approximate (isotropic) treatment of cell esds is used for estimating esds involving l.s. planes.

### Poly[[heptadecaaquatetradecaoxidononasodium] [pentacosaaquanitratoundecaoxidopentadecavanadium]] Fractional atomic coordinates and isotropic or equivalent isotropic displacement parameters (Å^2^)

**Table:**

	*x*	*y*	*z*	*U*_iso_*/*U*_eq_	
V2	0.20677 (7)	0.77632 (4)	0.38669 (4)	0.00758 (16)	
V6	0.53225 (7)	0.66034 (4)	0.51758 (4)	0.00747 (16)	
V4	0.28316 (7)	0.73693 (4)	0.51617 (4)	0.00731 (16)	
V3	0.59764 (7)	0.66385 (4)	0.38949 (4)	0.00760 (16)	
V5	0.29248 (7)	0.57664 (4)	0.52126 (4)	0.00765 (16)	
V1	0.22937 (7)	0.52768 (4)	0.39459 (4)	0.00770 (17)	
V7	0.47465 (7)	0.77731 (4)	0.45834 (4)	0.00759 (16)	
V8	0.48469 (7)	0.65619 (4)	0.26258 (4)	0.00814 (17)	
V9	0.45795 (7)	0.53686 (4)	0.32350 (4)	0.00823 (17)	
V10	0.11723 (7)	0.65526 (4)	0.46344 (4)	0.00850 (17)	
V11	0.09381 (7)	0.64984 (4)	0.31105 (4)	0.00995 (17)	
V12	0.23415 (7)	0.73086 (4)	0.25981 (4)	0.00950 (17)	
V13	0.24348 (7)	0.57055 (4)	0.26526 (4)	0.00873 (17)	
V14	0.47979 (7)	0.54602 (4)	0.47609 (4)	0.00808 (16)	
V15	0.44344 (7)	0.77200 (4)	0.30694 (4)	0.00878 (17)	
Na1	0.35864 (18)	0.98392 (10)	0.46670 (10)	0.0135 (4)	
Na4	0.83295 (18)	0.82583 (10)	0.31337 (10)	0.0138 (4)	
Na8	0.81651 (18)	0.51432 (10)	0.81930 (10)	0.0142 (4)	
Na5	0.85564 (18)	0.82460 (10)	0.46431 (10)	0.0140 (4)	
Na6	0.79182 (19)	0.70963 (11)	0.60608 (11)	0.0166 (5)	
Na3	0.67413 (18)	0.93001 (10)	0.17681 (10)	0.0146 (4)	
Na9	0.6930 (2)	0.40612 (11)	0.66796 (11)	0.0185 (5)	
Na2	0.4646 (2)	0.87886 (11)	0.59224 (12)	0.0207 (5)	
Na7	0.6663 (2)	0.59916 (13)	0.66662 (13)	0.0273 (6)	
O0P	0.3368 (3)	0.51518 (16)	0.46859 (17)	0.0087 (7)	
H0P	0.332486	0.474935	0.487152	0.013*	
O36	0.1701 (3)	0.57439 (15)	0.45978 (17)	0.0080 (7)	
H36	0.117419	0.548398	0.477163	0.012*	
O17	0.5584 (3)	0.71556 (16)	0.31636 (17)	0.0095 (7)	
H17	0.621713	0.730983	0.298531	0.014*	
O2	0.2527 (3)	0.67924 (16)	0.38662 (19)	0.0116 (7)	
O18	0.4895 (3)	0.80628 (16)	0.38474 (17)	0.0101 (7)	
H18	0.539 (4)	0.833 (2)	0.3884 (13)	0.015*	
O13	0.1232 (3)	0.73523 (16)	0.31424 (17)	0.0100 (7)	
H13	0.061997	0.759282	0.296068	0.015*	
O5	0.3156 (3)	0.51187 (16)	0.32311 (17)	0.0097 (7)	
H5	0.305302	0.470637	0.307036	0.015*	
O9	0.5858 (3)	0.72002 (16)	0.46109 (16)	0.0083 (7)	
H9	0.655205	0.736795	0.478056	0.012*	
O19	0.3254 (3)	0.78853 (16)	0.45131 (17)	0.0097 (7)	
H19	0.315708	0.829521	0.466911	0.015*	
O27	0.5006 (3)	0.51074 (16)	0.39777 (17)	0.0113 (7)	
H27	0.543 (4)	0.481 (2)	0.3950 (13)	0.017*	
O10	0.4333 (3)	0.72134 (16)	0.52258 (17)	0.0090 (7)	
H10	0.459786	0.741617	0.562139	0.013*	
O28	0.5717 (3)	0.60921 (15)	0.45589 (17)	0.0091 (7)	
H28	0.643270	0.595305	0.472584	0.014*	
O21	0.0440 (3)	0.65162 (16)	0.39056 (18)	0.0101 (7)	
H21	−0.0228 (11)	0.652 (3)	0.3955 (13)	0.015*	
O12	0.1503 (3)	0.73732 (16)	0.46030 (17)	0.0095 (7)	
H12	0.095685	0.762252	0.477178	0.014*	
O7	0.5510 (3)	0.60387 (15)	0.32675 (16)	0.0085 (7)	
H7	0.616388	0.589267	0.310617	0.013*	
O34	0.2964 (3)	0.78676 (16)	0.31905 (17)	0.0094 (7)	
H34	0.279161	0.826717	0.301067	0.014*	
O015	0.5221 (3)	0.82924 (16)	0.50296 (18)	0.0116 (7)	
O8	0.7248 (3)	0.66167 (16)	0.39076 (18)	0.0117 (7)	
O20	0.1455 (3)	0.83923 (17)	0.38674 (19)	0.0135 (8)	
O29	0.5498 (3)	0.50317 (17)	0.52288 (18)	0.0127 (8)	
O56	0.7149 (3)	0.76343 (18)	0.25700 (19)	0.0158 (8)	
H56A	0.673826	0.747454	0.281670	0.024*	
H56B	0.673194	0.784271	0.231168	0.024*	
O4	0.1660 (3)	0.46544 (16)	0.39460 (18)	0.0120 (7)	
O50	1.0132 (3)	0.80163 (17)	0.51996 (19)	0.0148 (8)	
H50A	1.042488	0.772381	0.502079	0.022*	
H50B	1.001935	0.787667	0.556089	0.022*	
O3	0.4248 (3)	0.68910 (16)	0.38798 (19)	0.0117 (7)	
O1	0.3555 (3)	0.60027 (16)	0.39778 (18)	0.0119 (7)	
O01E	0.3224 (3)	0.89538 (17)	0.51509 (19)	0.0146 (8)	
O52	0.9420 (3)	0.88225 (17)	0.3915 (2)	0.0158 (8)	
O25	0.3868 (3)	0.59093 (16)	0.26042 (17)	0.0110 (7)	
H25	0.400452	0.570697	0.221164	0.017*	
O53	0.7083 (3)	0.84455 (17)	0.3880 (2)	0.0153 (8)	
O55	0.8211 (3)	0.91159 (17)	0.24873 (19)	0.0141 (8)	
O45	0.9424 (3)	0.56896 (17)	0.88644 (19)	0.0143 (8)	
O23	0.1509 (3)	0.57220 (15)	0.32846 (17)	0.0095 (7)	
H23	0.092614	0.544963	0.312664	0.014*	
O51	0.8830 (3)	0.74821 (17)	0.3888 (2)	0.0157 (8)	
O48	0.8195 (3)	0.65100 (18)	0.5183 (2)	0.0159 (8)	
H48A	0.886410	0.648700	0.514941	0.024*	
H48B	0.793356	0.669046	0.484662	0.024*	
O58	0.5902 (3)	0.92532 (17)	0.26761 (18)	0.0146 (8)	
H58A	0.632427	0.938270	0.298757	0.022*	
H58B	0.579324	0.888487	0.276569	0.022*	
O33	0.0298 (3)	0.65191 (16)	0.51117 (19)	0.0121 (8)	
O01P	0.3771 (3)	1.06567 (17)	0.39712 (19)	0.0155 (8)	
O22	−0.0134 (3)	0.64033 (17)	0.26662 (19)	0.0146 (8)	
O32	0.2455 (3)	0.65614 (15)	0.52130 (18)	0.0113 (7)	
H32	0.214104	0.656642	0.561461	0.017*	
O54	0.9579 (3)	0.78898 (19)	0.2481 (2)	0.0187 (9)	
H54A	1.021132	0.791064	0.267651	0.028*	
H54B	0.959711	0.812342	0.216250	0.028*	
O40	0.6328 (3)	0.39548 (17)	0.56340 (19)	0.0162 (8)	
H40A	0.591280	0.365022	0.558594	0.024*	
H40B	0.685022	0.386610	0.542654	0.024*	
O01U	0.4953 (3)	0.78232 (19)	0.63590 (19)	0.0182 (9)	
H01A	0.487727	0.755881	0.606583	0.027*	
H01B	0.560799	0.779270	0.651182	0.027*	
O30	0.4289 (3)	0.60535 (16)	0.53324 (17)	0.0091 (7)	
H30	0.455438	0.589594	0.575060	0.014*	
O01W	0.9067 (4)	0.9561 (2)	0.6364 (2)	0.0215 (9)	
H01C	0.909158	0.939583	0.600385	0.032*	
H01D	0.870957	0.988345	0.628432	0.032*	
O44	0.9421 (3)	0.48703 (19)	0.75360 (19)	0.0170 (8)	
H44A	0.968283	0.452273	0.764512	0.026*	
H44B	0.996114	0.510993	0.759133	0.026*	
O59	0.7308 (4)	0.42783 (19)	0.7766 (2)	0.0185 (9)	
O6	0.5113 (3)	0.49090 (17)	0.27975 (18)	0.0135 (8)	
O49	0.7387 (3)	0.78079 (18)	0.5321 (2)	0.0174 (8)	
O60	0.5108 (3)	0.92596 (18)	0.1130 (2)	0.0174 (8)	
H60A	0.461797	0.914685	0.135294	0.026*	
H60B	0.512987	0.898067	0.085574	0.026*	
O022	0.2704 (3)	1.03401 (19)	0.5447 (2)	0.0209 (9)	
H02A	0.293614	1.070311	0.548999	0.031*	
H02B	0.288048	1.017771	0.580820	0.031*	
O023	0.2025 (3)	0.96462 (17)	0.39936 (19)	0.0152 (8)	
O47	0.9660 (3)	0.74376 (18)	0.62561 (19)	0.0174 (8)	
H47A	0.995484	0.744417	0.591022	0.026*	
H47B	1.003483	0.718068	0.648833	0.026*	
O025	0.5276 (3)	1.00113 (19)	0.5126 (2)	0.0187 (9)	
H02C	0.526138	1.027720	0.541404	0.028*	
H02D	0.551393	0.969512	0.532185	0.028*	
O15	0.3738 (3)	0.71159 (16)	0.25203 (18)	0.0117 (7)	
H15	0.382663	0.728191	0.210257	0.018*	
O46	0.8429 (3)	0.62287 (19)	0.6648 (2)	0.0180 (8)	
O14	0.1926 (3)	0.76448 (17)	0.19616 (18)	0.0130 (8)	
H14	0.259 (3)	0.784 (2)	0.212 (2)	0.019*	
O43	0.7137 (4)	0.5983 (2)	0.7750 (2)	0.0278 (11)	
O41	0.8037 (3)	0.32520 (18)	0.69269 (19)	0.0159 (8)	
H41A	0.805479	0.303061	0.660055	0.024*	
H41B	0.776454	0.302712	0.719266	0.024*	
O24	0.2020 (3)	0.65165 (16)	0.25263 (18)	0.0121 (8)	
H24	0.158570	0.649984	0.211531	0.018*	
O16	0.5415 (3)	0.65230 (17)	0.19990 (19)	0.0140 (8)	
H16	0.581 (4)	0.621 (2)	0.2259 (17)	0.021*	
O35	0.4751 (3)	0.82263 (17)	0.26029 (18)	0.0123 (7)	
O31	0.2654 (3)	0.54706 (17)	0.58524 (18)	0.0118 (7)	
H31	0.306 (5)	0.5803 (19)	0.5995 (12)	0.018*	
O37	0.6175 (3)	0.66028 (17)	0.57800 (18)	0.0122 (8)	
O02G	0.8149 (4)	0.90770 (18)	0.5250 (2)	0.0204 (9)	
H02E	0.867950	0.915319	0.552518	0.031*	
H02F	0.764052	0.898575	0.546883	0.031*	
O39	0.6093 (4)	0.49960 (19)	0.6682 (2)	0.0229 (9)	
O38	0.4855 (4)	0.6140 (2)	0.6757 (2)	0.0238 (10)	
H38A	0.477395	0.646038	0.696937	0.036*	
H38B	0.462626	0.585768	0.698122	0.036*	
O02J	0.3616 (4)	0.95214 (19)	0.6379 (2)	0.0216 (9)	
O02K	0.6354 (4)	0.9172 (2)	0.5893 (2)	0.0235 (10)	
H02G	0.639660	0.952681	0.605813	0.035*	
H02H	0.646888	0.923140	0.550648	0.035*	
O11	0.2639 (3)	0.77014 (18)	0.57991 (18)	0.0135 (8)	
O57	0.6507 (4)	0.82705 (19)	0.1516 (2)	0.0192 (9)	
H57A	0.663709	0.806052	0.185109	0.029*	
H57B	0.584844	0.820373	0.139387	0.029*	
O26	0.1980 (3)	0.53413 (17)	0.20435 (18)	0.0129 (8)	
H26	0.235 (5)	0.5673 (19)	0.1828 (12)	0.019*	
N1	0.3448 (4)	0.6563 (2)	0.3907 (2)	0.0159 (10)	

### Poly[[heptadecaaquatetradecaoxidononasodium] [pentacosaaquanitratoundecaoxidopentadecavanadium]] Atomic displacement parameters (Å^2^)

**Table:**

	*U*^11^	*U*^22^	*U*^33^	*U*^12^	*U*^13^	*U*^23^
V2	0.0066 (4)	0.0077 (4)	0.0084 (4)	0.0009 (3)	0.0004 (3)	0.0000 (3)
V6	0.0068 (4)	0.0082 (4)	0.0073 (4)	−0.0002 (3)	0.0003 (3)	−0.0001 (3)
V4	0.0066 (4)	0.0086 (4)	0.0067 (4)	−0.0001 (3)	0.0008 (3)	−0.0008 (3)
V3	0.0059 (4)	0.0089 (4)	0.0080 (4)	−0.0002 (3)	0.0008 (3)	−0.0004 (3)
V5	0.0069 (4)	0.0085 (4)	0.0075 (4)	−0.0003 (3)	0.0008 (3)	0.0009 (3)
V1	0.0075 (4)	0.0068 (4)	0.0087 (4)	−0.0006 (3)	0.0003 (3)	0.0004 (3)
V7	0.0067 (4)	0.0075 (4)	0.0085 (4)	−0.0005 (3)	0.0005 (3)	−0.0004 (3)
V8	0.0084 (4)	0.0085 (4)	0.0077 (4)	−0.0003 (3)	0.0017 (3)	0.0005 (3)
V9	0.0087 (4)	0.0075 (4)	0.0086 (4)	0.0001 (3)	0.0010 (3)	−0.0005 (3)
V10	0.0064 (4)	0.0083 (4)	0.0110 (4)	0.0004 (3)	0.0016 (3)	0.0005 (3)
V11	0.0082 (4)	0.0086 (4)	0.0123 (4)	−0.0001 (3)	−0.0019 (3)	0.0008 (3)
V12	0.0100 (4)	0.0097 (4)	0.0086 (4)	−0.0002 (3)	0.0001 (3)	0.0010 (3)
V13	0.0091 (4)	0.0091 (4)	0.0079 (4)	−0.0005 (3)	0.0002 (3)	−0.0009 (3)
V14	0.0068 (4)	0.0078 (4)	0.0093 (4)	0.0003 (3)	−0.0003 (3)	0.0016 (3)
V15	0.0079 (4)	0.0094 (4)	0.0092 (4)	0.0002 (3)	0.0012 (3)	0.0016 (3)
Na1	0.0127 (11)	0.0145 (10)	0.0131 (10)	−0.0027 (8)	0.0012 (9)	−0.0006 (8)
Na4	0.0121 (11)	0.0131 (10)	0.0161 (11)	−0.0007 (8)	0.0014 (9)	0.0006 (8)
Na8	0.0141 (11)	0.0154 (10)	0.0134 (10)	−0.0004 (8)	0.0020 (9)	−0.0011 (8)
Na5	0.0114 (11)	0.0145 (10)	0.0158 (11)	0.0007 (8)	−0.0006 (9)	0.0005 (8)
Na6	0.0146 (12)	0.0203 (11)	0.0149 (11)	0.0026 (9)	0.0012 (9)	0.0018 (9)
Na3	0.0127 (11)	0.0175 (11)	0.0135 (10)	−0.0021 (8)	0.0012 (9)	0.0002 (8)
Na9	0.0191 (12)	0.0209 (12)	0.0158 (11)	0.0019 (9)	0.0026 (10)	0.0001 (9)
Na2	0.0205 (13)	0.0194 (12)	0.0220 (12)	0.0042 (10)	0.0012 (10)	−0.0007 (9)
Na7	0.0214 (14)	0.0350 (15)	0.0250 (13)	−0.0045 (11)	−0.0006 (11)	0.0115 (11)
O0P	0.0055 (17)	0.0105 (17)	0.0098 (16)	0.0007 (13)	−0.0004 (14)	0.0031 (13)
O36	0.0075 (17)	0.0080 (16)	0.0088 (16)	−0.0003 (13)	0.0015 (14)	0.0013 (13)
O17	0.0080 (18)	0.0094 (16)	0.0114 (17)	−0.0002 (13)	0.0024 (14)	0.0000 (13)
O2	0.0066 (18)	0.0093 (16)	0.0191 (19)	0.0007 (14)	0.0016 (15)	0.0016 (15)
O18	0.0085 (18)	0.0090 (17)	0.0132 (18)	−0.0010 (13)	0.0022 (15)	−0.0011 (14)
O13	0.0084 (18)	0.0094 (16)	0.0113 (17)	−0.0002 (13)	−0.0029 (14)	0.0011 (13)
O5	0.0089 (18)	0.0087 (16)	0.0118 (17)	−0.0005 (13)	0.0024 (14)	−0.0005 (13)
O9	0.0060 (17)	0.0093 (16)	0.0095 (17)	0.0002 (13)	0.0011 (14)	−0.0013 (13)
O19	0.0080 (18)	0.0096 (16)	0.0113 (17)	−0.0006 (13)	0.0004 (14)	0.0000 (13)
O27	0.0112 (19)	0.0110 (17)	0.0120 (18)	−0.0010 (14)	0.0028 (15)	0.0012 (14)
O10	0.0080 (18)	0.0089 (16)	0.0101 (17)	−0.0001 (13)	0.0009 (14)	−0.0002 (13)
O28	0.0070 (17)	0.0103 (16)	0.0100 (16)	0.0012 (13)	0.0018 (14)	0.0012 (13)
O21	0.0064 (17)	0.0109 (17)	0.0129 (18)	0.0015 (13)	0.0011 (14)	0.0001 (13)
O12	0.0078 (18)	0.0098 (16)	0.0109 (17)	0.0009 (13)	0.0019 (14)	−0.0003 (13)
O7	0.0094 (18)	0.0096 (16)	0.0060 (15)	0.0006 (13)	−0.0013 (14)	−0.0009 (13)
O34	0.0103 (18)	0.0076 (16)	0.0098 (17)	−0.0006 (13)	−0.0009 (14)	0.0008 (13)
O015	0.0117 (19)	0.0098 (16)	0.0132 (18)	−0.0006 (14)	0.0012 (15)	0.0005 (14)
O8	0.0103 (19)	0.0130 (17)	0.0119 (18)	0.0007 (14)	0.0017 (15)	−0.0002 (14)
O20	0.013 (2)	0.0128 (18)	0.0150 (19)	−0.0003 (15)	0.0029 (16)	−0.0010 (14)
O29	0.0118 (19)	0.0113 (17)	0.0151 (19)	0.0009 (14)	0.0014 (15)	0.0018 (14)
O56	0.013 (2)	0.018 (2)	0.017 (2)	−0.0012 (15)	0.0042 (16)	0.0003 (16)
O4	0.0115 (19)	0.0108 (17)	0.0139 (18)	−0.0006 (14)	0.0014 (15)	0.0006 (14)
O50	0.013 (2)	0.0152 (19)	0.0167 (19)	0.0033 (15)	0.0040 (16)	−0.0016 (15)
O3	0.0077 (18)	0.0090 (17)	0.0182 (19)	−0.0007 (13)	0.0013 (15)	−0.0003 (14)
O1	0.0096 (18)	0.0078 (16)	0.0182 (19)	0.0010 (14)	0.0008 (15)	0.0000 (14)
O01E	0.015 (2)	0.0101 (17)	0.019 (2)	−0.0008 (15)	0.0051 (16)	−0.0013 (15)
O52	0.012 (2)	0.0134 (18)	0.022 (2)	−0.0022 (15)	−0.0003 (17)	0.0021 (16)
O25	0.0128 (19)	0.0085 (17)	0.0112 (18)	−0.0002 (14)	−0.0011 (15)	0.0006 (13)
O53	0.012 (2)	0.0153 (19)	0.019 (2)	−0.0017 (15)	0.0025 (16)	−0.0015 (15)
O55	0.0127 (19)	0.0123 (18)	0.0167 (19)	0.0015 (15)	−0.0010 (16)	−0.0027 (15)
O45	0.015 (2)	0.0101 (17)	0.0174 (19)	−0.0008 (14)	0.0015 (16)	0.0009 (15)
O23	0.0106 (18)	0.0074 (16)	0.0106 (17)	−0.0011 (13)	0.0017 (14)	0.0005 (13)
O51	0.014 (2)	0.0133 (18)	0.019 (2)	0.0021 (15)	−0.0011 (17)	0.0014 (15)
O48	0.0097 (19)	0.022 (2)	0.016 (2)	0.0019 (15)	0.0022 (16)	0.0004 (16)
O58	0.016 (2)	0.0142 (19)	0.0139 (19)	−0.0010 (15)	0.0019 (16)	0.0019 (15)
O33	0.0079 (19)	0.0139 (18)	0.0142 (19)	−0.0005 (14)	0.0004 (15)	0.0022 (14)
O01P	0.014 (2)	0.0132 (18)	0.019 (2)	0.0001 (15)	0.0011 (17)	0.0000 (15)
O22	0.013 (2)	0.0135 (18)	0.0169 (19)	−0.0015 (15)	−0.0019 (16)	0.0003 (15)
O32	0.0085 (18)	0.0093 (17)	0.0160 (19)	0.0010 (13)	0.0007 (15)	0.0012 (14)
O54	0.010 (2)	0.022 (2)	0.023 (2)	0.0046 (16)	0.0001 (17)	0.0039 (17)
O40	0.017 (2)	0.0108 (18)	0.021 (2)	−0.0001 (15)	0.0034 (17)	−0.0008 (15)
O01U	0.016 (2)	0.024 (2)	0.014 (2)	0.0000 (16)	0.0000 (16)	−0.0029 (16)
O30	0.0093 (18)	0.0090 (16)	0.0089 (17)	0.0003 (13)	0.0000 (14)	−0.0013 (13)
O01W	0.022 (2)	0.025 (2)	0.017 (2)	0.0073 (18)	0.0006 (18)	0.0061 (17)
O44	0.014 (2)	0.0165 (19)	0.021 (2)	0.0010 (15)	0.0040 (17)	0.0038 (16)
O59	0.020 (2)	0.020 (2)	0.016 (2)	−0.0067 (17)	0.0038 (17)	−0.0044 (16)
O6	0.013 (2)	0.0138 (18)	0.0134 (19)	−0.0006 (15)	0.0019 (15)	−0.0002 (14)
O49	0.018 (2)	0.0133 (19)	0.020 (2)	−0.0026 (16)	−0.0013 (17)	0.0032 (16)
O60	0.011 (2)	0.020 (2)	0.021 (2)	0.0001 (15)	0.0027 (17)	−0.0015 (16)
O022	0.018 (2)	0.018 (2)	0.026 (2)	−0.0013 (17)	0.0001 (18)	0.0020 (17)
O023	0.0106 (19)	0.0159 (19)	0.019 (2)	0.0016 (15)	0.0008 (16)	0.0010 (15)
O47	0.020 (2)	0.019 (2)	0.0128 (19)	0.0031 (16)	0.0002 (17)	−0.0004 (15)
O025	0.017 (2)	0.021 (2)	0.018 (2)	−0.0033 (17)	0.0008 (17)	0.0003 (17)
O15	0.0104 (19)	0.0109 (17)	0.0137 (18)	−0.0015 (14)	0.0001 (15)	0.0026 (14)
O46	0.016 (2)	0.020 (2)	0.018 (2)	0.0014 (16)	0.0014 (17)	0.0010 (16)
O14	0.0103 (19)	0.0165 (19)	0.0117 (18)	0.0002 (15)	−0.0004 (15)	0.0038 (14)
O43	0.041 (3)	0.023 (2)	0.019 (2)	0.006 (2)	0.001 (2)	0.0012 (18)
O41	0.014 (2)	0.0193 (19)	0.0144 (19)	−0.0021 (16)	0.0034 (16)	−0.0011 (16)
O24	0.0119 (19)	0.0108 (17)	0.0131 (18)	−0.0007 (14)	−0.0012 (15)	0.0003 (14)
O16	0.014 (2)	0.0160 (19)	0.0129 (19)	0.0024 (15)	0.0042 (16)	0.0024 (14)
O35	0.0121 (19)	0.0121 (17)	0.0120 (18)	0.0002 (15)	−0.0019 (15)	0.0018 (14)
O31	0.0104 (19)	0.0120 (18)	0.0128 (18)	−0.0002 (14)	0.0003 (15)	0.0012 (14)
O37	0.0093 (19)	0.0155 (19)	0.0113 (18)	−0.0003 (14)	−0.0005 (15)	0.0020 (14)
O02G	0.026 (2)	0.015 (2)	0.020 (2)	−0.0013 (17)	0.0044 (19)	0.0002 (16)
O39	0.019 (2)	0.018 (2)	0.032 (2)	0.0033 (17)	0.0029 (19)	−0.0036 (18)
O38	0.030 (3)	0.021 (2)	0.022 (2)	−0.0037 (18)	0.011 (2)	0.0013 (18)
O02J	0.025 (2)	0.018 (2)	0.022 (2)	0.0014 (17)	0.0029 (19)	0.0001 (17)
O02K	0.026 (2)	0.022 (2)	0.021 (2)	−0.0063 (18)	−0.0014 (19)	−0.0031 (17)
O11	0.0098 (19)	0.0176 (19)	0.0134 (19)	−0.0004 (15)	0.0025 (15)	−0.0027 (15)
O57	0.017 (2)	0.022 (2)	0.019 (2)	−0.0014 (17)	0.0036 (17)	−0.0042 (17)
O26	0.015 (2)	0.0124 (18)	0.0107 (18)	−0.0018 (15)	−0.0006 (15)	−0.0002 (14)
N1	0.017 (3)	0.016 (2)	0.014 (2)	−0.0001 (19)	0.003 (2)	−0.0014 (18)

### Poly[[heptadecaaquatetradecaoxidononasodium] [pentacosaaquanitratoundecaoxidopentadecavanadium]] Geometric parameters (Å, º)

**Table:**

V2—V4	3.0099 (13)	Na4—O55	2.390 (5)
V2—V12	2.9975 (12)	Na4—O51	2.439 (5)
V2—O2	2.274 (4)	Na4—O54	2.402 (5)
V2—O13	2.030 (4)	Na8—Na7	4.122 (4)
V2—O19	1.973 (4)	Na8—O45	2.397 (5)
V2—O12	2.028 (4)	Na8—O01P^ii^	2.540 (5)
V2—O34	1.976 (4)	Na8—O44	2.348 (5)
V2—O20	1.626 (4)	Na8—O59	2.382 (5)
V6—V3	2.9929 (12)	Na8—O023^ii^	2.438 (4)
V6—V7	2.9997 (12)	Na8—O43	2.449 (5)
V6—V14	2.7971 (12)	Na5—Na6	4.180 (3)
V6—O9	1.996 (4)	Na5—O50	2.298 (5)
V6—O10	1.886 (4)	Na5—O52	2.410 (5)
V6—O28	1.881 (4)	Na5—O53	2.416 (5)
V6—O30	1.876 (4)	Na5—O51	2.435 (5)
V6—O37	1.614 (4)	Na5—O49	2.425 (5)
V4—V7	3.0197 (12)	Na5—O02G	2.387 (5)
V4—V10	2.9534 (13)	Na6—Na7	3.321 (4)
V4—O19	1.952 (4)	Na6—O48	2.381 (5)
V4—O10	1.948 (4)	Na6—O49	2.324 (5)
V4—O12	1.980 (4)	Na6—O47	2.358 (5)
V4—O32	1.897 (4)	Na6—O46	2.393 (5)
V4—O11	1.618 (4)	Na6—O14^ii^	2.517 (4)
V3—V8	2.9750 (13)	Na6—O37	2.514 (5)
V3—O17	1.992 (4)	Na3—O55	2.350 (5)
V3—O9	2.027 (4)	Na3—O58	2.350 (4)
V3—O28	1.955 (4)	Na3—O60	2.384 (5)
V3—O7	1.969 (4)	Na3—O31^iii^	2.471 (4)
V3—O8	1.628 (4)	Na3—O57	2.405 (5)
V3—O3	2.286 (4)	Na3—O26^iv^	2.442 (4)
V5—V1	2.9938 (13)	Na9—Na2^v^	4.058 (3)
V5—V10	3.0291 (13)	Na9—O40	2.330 (5)
V5—V14	2.7801 (12)	Na9—O59	2.406 (5)
V5—O0P	1.926 (4)	Na9—O41	2.343 (5)
V5—O36	1.947 (4)	Na9—O16^vi^	2.510 (5)
V5—O32	1.897 (4)	Na9—H16^vi^	2.10 (3)
V5—O30	1.858 (4)	Na9—O39	2.373 (5)
V5—O31	1.614 (4)	Na9—O02J^v^	2.549 (5)
V5—H31	1.69 (3)	Na2—O015	2.424 (4)
V1—V13	2.9955 (12)	Na2—O01E	2.367 (5)
V1—O0P	2.019 (4)	Na2—O01U	2.397 (5)
V1—O36	1.984 (4)	Na2—O02J	2.400 (5)
V1—O5	2.034 (4)	Na2—O02K	2.364 (5)
V1—O4	1.626 (4)	Na7—O46	2.331 (5)
V1—O1	2.301 (4)	Na7—O43	2.365 (5)
V1—O23	1.943 (4)	Na7—O37	2.397 (5)
V7—O18	1.757 (4)	Na7—O39	2.370 (5)
V7—O9	1.923 (4)	Na7—O38	2.373 (6)
V7—O19	1.920 (4)	O0P—H0P	1.0000
V7—O10	1.998 (4)	O36—H36	1.0000
V7—O015	1.600 (4)	O17—H17	1.0000
V8—V9	3.0428 (12)	O2—N1	1.284 (6)
V8—V15	2.8611 (12)	O18—H18	0.873 (13)
V8—O17	1.955 (4)	O13—H13	1.0000
V8—O7	1.951 (4)	O5—H5	1.0000
V8—O25	1.936 (4)	O9—H9	1.0000
V8—O15	1.891 (4)	O19—H19	1.0000
V8—O16	1.615 (4)	O27—H27	0.873 (13)
V8—H16	1.74 (3)	O10—H10	1.0000
V9—V13	2.9977 (13)	O28—H28	1.0000
V9—O5	1.910 (4)	O21—H21	0.875 (13)
V9—O27	1.748 (4)	O12—H12	1.0000
V9—O7	1.926 (4)	O7—H7	1.0000
V9—O25	1.983 (4)	O34—H34	1.0000
V9—O6	1.611 (4)	O56—H56A	0.8703
V10—O36	1.956 (4)	O56—H56B	0.8706
V10—O21	1.752 (4)	O50—H50A	0.8729
V10—O12	1.908 (4)	O50—H50B	0.8724
V10—O33	1.609 (4)	O3—N1	1.273 (6)
V10—O32	1.958 (4)	O1—N1	1.282 (6)
V11—V12	2.8771 (13)	O25—H25	1.0000
V11—V13	2.8827 (13)	O23—H23	1.0000
V11—O13	1.968 (4)	O48—H48A	0.8710
V11—O21	1.906 (4)	O48—H48B	0.8708
V11—O23	1.925 (4)	O58—H58A	0.8700
V11—O22	1.606 (4)	O58—H58B	0.8705
V11—O24	1.981 (4)	O32—H32	1.0000
V12—V15	2.9186 (13)	O54—H54A	0.8747
V12—O13	1.950 (4)	O54—H54B	0.8727
V12—O34	1.915 (4)	O40—H40A	0.8701
V12—O15	1.868 (4)	O40—H40B	0.8709
V12—O14	1.616 (4)	O01U—H01A	0.8714
V12—H14	1.65 (3)	O01U—H01B	0.8713
V12—O24	1.842 (4)	O30—H30	1.0000
V13—O5	1.986 (4)	O01W—H01C	0.8701
V13—O25	1.909 (4)	O01W—H01D	0.8691
V13—O23	1.911 (4)	O44—H44A	0.8776
V13—O24	1.922 (4)	O44—H44B	0.8775
V13—O26	1.612 (4)	O60—H60A	0.8714
V13—H26	1.78 (3)	O60—H60B	0.8710
V14—O0P	1.952 (4)	O022—H02A	0.8751
V14—O27	1.924 (4)	O022—H02B	0.8740
V14—O28	1.934 (4)	O47—H47A	0.8770
V14—O29	1.606 (4)	O47—H47B	0.8774
V14—O30	1.986 (4)	O025—H02C	0.8703
V15—O17	1.944 (4)	O025—H02D	0.8706
V15—O18	1.894 (4)	O15—H15	1.0000
V15—O34	1.961 (4)	O14—H14	0.993 (14)
V15—O15	1.964 (4)	O41—H41A	0.8703
V15—O35	1.611 (4)	O41—H41B	0.8710
Na1—Na8^i^	3.188 (3)	O24—H24	1.0000
Na1—Na2	3.758 (3)	O16—H16	1.009 (14)
Na1—O01E	2.333 (4)	O31—H31	0.948 (14)
Na1—O45^i^	2.457 (4)	O02G—H02E	0.8721
Na1—O01P	2.416 (5)	O02G—H02F	0.8722
Na1—O022	2.418 (5)	O38—H38A	0.8713
Na1—O023	2.387 (5)	O38—H38B	0.8725
Na1—O025	2.317 (5)	O02K—H02G	0.8789
Na4—Na5	3.259 (3)	O02K—H02H	0.8779
Na4—Na3	4.145 (3)	O57—H57A	0.8699
Na4—O56	2.319 (5)	O57—H57B	0.8704
Na4—O52	2.439 (5)	O26—H26	1.027 (14)
Na4—O53	2.434 (5)		
			
V12—V2—V4	134.29 (4)	O022—Na1—Na8^i^	131.01 (14)
O2—V2—V4	69.92 (11)	O022—Na1—Na2	87.04 (13)
O2—V2—V12	67.14 (10)	O022—Na1—O45^i^	177.94 (18)
O13—V2—V4	132.75 (11)	O023—Na1—Na8^i^	49.33 (11)
O13—V2—V12	40.13 (11)	O023—Na1—Na2	122.77 (13)
O13—V2—O2	70.85 (15)	O023—Na1—O45^i^	83.03 (15)
O19—V2—V4	39.66 (11)	O023—Na1—O01P	83.67 (16)
O19—V2—V12	122.89 (11)	O023—Na1—O022	95.14 (16)
O19—V2—O2	87.48 (15)	O025—Na1—Na8^i^	118.51 (14)
O19—V2—O13	156.37 (16)	O025—Na1—Na2	63.10 (12)
O19—V2—O12	79.29 (15)	O025—Na1—O01E	100.00 (17)
O19—V2—O34	92.63 (15)	O025—Na1—O45^i^	85.74 (16)
O12—V2—V4	40.74 (11)	O025—Na1—O01P	89.39 (16)
O12—V2—V12	131.54 (11)	O025—Na1—O022	96.17 (17)
O12—V2—O2	72.25 (14)	O025—Na1—O023	167.57 (18)
O12—V2—O13	102.00 (16)	Na5—Na4—Na3	135.02 (8)
O34—V2—V4	125.00 (12)	O56—Na4—Na5	120.47 (14)
O34—V2—V12	38.88 (11)	O56—Na4—Na3	74.17 (12)
O34—V2—O2	86.74 (14)	O56—Na4—O52	167.86 (17)
O34—V2—O13	77.26 (15)	O56—Na4—O53	91.10 (16)
O34—V2—O12	157.67 (15)	O56—Na4—O55	100.86 (17)
O20—V2—V4	111.51 (15)	O56—Na4—O51	91.41 (16)
O20—V2—V12	113.97 (15)	O56—Na4—O54	85.19 (16)
O20—V2—O2	166.11 (18)	O52—Na4—Na5	47.40 (11)
O20—V2—O13	100.79 (19)	O52—Na4—Na3	113.57 (12)
O20—V2—O19	102.25 (19)	O53—Na4—Na5	47.54 (12)
O20—V2—O12	99.57 (18)	O53—Na4—Na3	94.03 (12)
O20—V2—O34	102.50 (18)	O53—Na4—O52	79.28 (15)
V3—V6—V7	70.06 (3)	O53—Na4—O51	79.76 (15)
V14—V6—V3	78.88 (3)	O55—Na4—Na5	126.20 (13)
V14—V6—V7	129.69 (4)	O55—Na4—Na3	28.76 (11)
O9—V6—V3	42.33 (10)	O55—Na4—O52	88.77 (15)
O9—V6—V7	39.17 (10)	O55—Na4—O53	104.21 (16)
O9—V6—V14	120.97 (11)	O55—Na4—O51	166.95 (18)
O10—V6—V3	107.02 (12)	O55—Na4—O54	86.49 (16)
O10—V6—V7	40.82 (11)	O51—Na4—Na5	47.98 (11)
O10—V6—V14	123.77 (12)	O51—Na4—Na3	164.28 (13)
O10—V6—O9	79.58 (15)	O51—Na4—O52	79.69 (16)
O28—V6—V3	39.62 (11)	O54—Na4—Na5	126.78 (14)
O28—V6—V7	108.07 (12)	O54—Na4—Na3	94.73 (13)
O28—V6—V14	43.59 (11)	O54—Na4—O52	102.92 (17)
O28—V6—O9	80.85 (15)	O54—Na4—O53	169.18 (17)
O28—V6—O10	136.58 (17)	O54—Na4—O51	90.16 (16)
O30—V6—V3	117.48 (12)	Na1^ii^—Na8—Na7	144.58 (9)
O30—V6—V7	121.15 (12)	O45—Na8—Na1^ii^	49.75 (11)
O30—V6—V14	45.18 (11)	O45—Na8—Na7	118.26 (13)
O30—V6—O9	149.05 (17)	O45—Na8—O01P^ii^	79.99 (15)
O30—V6—O10	88.97 (16)	O45—Na8—O023^ii^	83.22 (15)
O30—V6—O28	88.37 (16)	O45—Na8—O43	97.88 (17)
O37—V6—V3	121.46 (14)	O01P^ii^—Na8—Na1^ii^	48.29 (11)
O37—V6—V7	117.57 (14)	O01P^ii^—Na8—Na7	161.67 (13)
O37—V6—V14	112.26 (14)	O44—Na8—Na1^ii^	124.14 (14)
O37—V6—O9	104.61 (18)	O44—Na8—Na7	85.70 (13)
O37—V6—O10	110.74 (18)	O44—Na8—O45	92.52 (16)
O37—V6—O28	111.56 (19)	O44—Na8—O01P^ii^	92.11 (16)
O37—V6—O30	106.34 (18)	O44—Na8—O59	82.27 (16)
V2—V4—V7	73.63 (3)	O44—Na8—O023^ii^	171.65 (18)
V10—V4—V2	71.09 (3)	O44—Na8—O43	109.93 (18)
V10—V4—V7	127.76 (4)	O59—Na8—Na1^ii^	114.93 (14)
O19—V4—V2	40.20 (11)	O59—Na8—Na7	85.09 (13)
O19—V4—V7	38.37 (11)	O59—Na8—O45	155.76 (18)
O19—V4—V10	109.87 (12)	O59—Na8—O01P^ii^	76.58 (16)
O19—V4—O12	80.98 (16)	O59—Na8—O023^ii^	98.69 (16)
O10—V4—V2	109.79 (11)	O59—Na8—O43	106.16 (19)
O10—V4—V7	40.69 (11)	O023^ii^—Na8—Na1^ii^	47.96 (11)
O10—V4—V10	125.40 (11)	O023^ii^—Na8—Na7	102.64 (13)
O10—V4—O19	79.01 (15)	O023^ii^—Na8—O01P^ii^	80.11 (15)
O10—V4—O12	145.29 (16)	O023^ii^—Na8—O43	77.85 (16)
O12—V4—V2	41.93 (11)	O43—Na8—Na1^ii^	114.20 (14)
O12—V4—V7	115.34 (11)	O43—Na8—Na7	30.50 (12)
O12—V4—V10	39.67 (11)	O43—Na8—O01P^ii^	157.96 (17)
O32—V4—V2	106.34 (13)	Na4—Na5—Na6	138.00 (8)
O32—V4—V7	122.67 (12)	O50—Na5—Na4	120.51 (13)
O32—V4—V10	40.76 (12)	O50—Na5—Na6	72.75 (12)
O32—V4—O19	135.35 (17)	O50—Na5—O52	91.52 (16)
O32—V4—O10	94.44 (16)	O50—Na5—O53	168.51 (17)
O32—V4—O12	80.38 (16)	O50—Na5—O51	90.61 (16)
O11—V4—V2	126.69 (15)	O50—Na5—O49	98.92 (17)
O11—V4—V7	114.98 (15)	O50—Na5—O02G	96.93 (17)
O11—V4—V10	116.86 (15)	O52—Na5—Na4	48.15 (11)
O11—V4—O19	114.83 (18)	O52—Na5—Na6	163.94 (14)
O11—V4—O10	105.37 (18)	O52—Na5—O53	80.21 (15)
O11—V4—O12	108.72 (18)	O52—Na5—O51	80.33 (15)
O11—V4—O32	109.51 (19)	O52—Na5—O49	168.03 (18)
V8—V3—V6	134.58 (4)	O53—Na5—Na4	48.02 (11)
O17—V3—V6	133.18 (11)	O53—Na5—Na6	114.86 (12)
O17—V3—V8	40.61 (11)	O53—Na5—O51	80.20 (15)
O17—V3—O9	102.09 (15)	O53—Na5—O49	90.21 (16)
O17—V3—O3	71.44 (15)	O51—Na5—Na4	48.08 (11)
O9—V3—V6	41.54 (10)	O51—Na5—Na6	96.24 (12)
O9—V3—V8	132.65 (11)	O49—Na5—Na4	128.37 (14)
O9—V3—O3	72.57 (15)	O49—Na5—Na6	27.70 (11)
O28—V3—V6	37.84 (11)	O49—Na5—O51	105.24 (16)
O28—V3—V8	122.79 (12)	O02G—Na5—Na4	123.23 (14)
O28—V3—O17	155.60 (16)	O02G—Na5—Na6	90.63 (12)
O28—V3—O9	78.33 (15)	O02G—Na5—O52	94.58 (16)
O28—V3—O7	90.64 (15)	O02G—Na5—O53	91.71 (17)
O28—V3—O3	85.75 (15)	O02G—Na5—O51	171.04 (18)
O7—V3—V6	122.00 (11)	O02G—Na5—O49	78.43 (16)
O7—V3—V8	40.41 (11)	Na7—Na6—Na5	154.49 (10)
O7—V3—O17	79.75 (15)	O48—Na6—Na5	72.46 (12)
O7—V3—O9	156.99 (16)	O48—Na6—Na7	91.07 (13)
O7—V3—O3	86.65 (15)	O48—Na6—O46	85.04 (16)
O8—V3—V6	111.42 (15)	O48—Na6—O14^ii^	152.17 (17)
O8—V3—V8	113.52 (15)	O48—Na6—O37	76.38 (15)
O8—V3—O17	101.33 (18)	O49—Na6—Na5	29.02 (12)
O8—V3—O9	99.48 (18)	O49—Na6—Na7	132.62 (15)
O8—V3—O28	102.66 (18)	O49—Na6—O48	83.70 (16)
O8—V3—O7	102.64 (18)	O49—Na6—O47	95.62 (17)
O8—V3—O3	167.25 (17)	O49—Na6—O46	168.35 (19)
O3—V3—V6	69.52 (10)	O49—Na6—O14^ii^	103.96 (16)
O3—V3—V8	68.17 (11)	O49—Na6—O37	87.38 (16)
V1—V5—V10	73.90 (3)	O47—Na6—Na5	70.77 (12)
V1—V5—H31	158.3 (7)	O47—Na6—Na7	131.75 (14)
V10—V5—H31	112 (2)	O47—Na6—O48	96.04 (16)
V14—V5—V1	74.98 (3)	O47—Na6—O46	88.52 (17)
V14—V5—V10	129.11 (4)	O47—Na6—O14^ii^	109.50 (16)
V14—V5—H31	112 (2)	O47—Na6—O37	171.53 (17)
O0P—V5—V1	41.81 (11)	O46—Na6—Na5	147.03 (13)
O0P—V5—V10	115.59 (12)	O46—Na6—Na7	44.58 (12)
O0P—V5—V14	44.58 (11)	O46—Na6—O14^ii^	84.78 (15)
O0P—V5—O36	80.92 (16)	O46—Na6—O37	87.08 (16)
O0P—V5—H31	129 (2)	O14^ii^—Na6—Na5	125.74 (12)
O36—V5—V1	40.85 (10)	O14^ii^—Na6—Na7	63.86 (11)
O36—V5—V10	39.21 (10)	O37—Na6—Na5	109.78 (12)
O36—V5—V14	114.49 (11)	O37—Na6—Na7	45.98 (11)
O36—V5—H31	133 (2)	O37—Na6—O14^ii^	77.29 (14)
O32—V5—V1	107.30 (13)	O55—Na3—Na4	29.30 (11)
O32—V5—V10	38.93 (12)	O55—Na3—O60	165.82 (18)
O32—V5—V14	121.55 (12)	O55—Na3—O31^iii^	98.97 (16)
O32—V5—O0P	143.09 (17)	O55—Na3—O57	92.90 (17)
O32—V5—O36	78.05 (16)	O55—Na3—O26^iv^	86.46 (15)
O32—V5—H31	87 (2)	O58—Na3—Na4	66.94 (12)
O30—V5—V1	114.45 (12)	O58—Na3—O55	80.92 (16)
O30—V5—V10	119.84 (12)	O58—Na3—O60	91.80 (16)
O30—V5—V14	45.54 (11)	O58—Na3—O31^iii^	170.32 (17)
O30—V5—O0P	90.05 (16)	O58—Na3—O57	95.16 (16)
O30—V5—O36	141.41 (16)	O58—Na3—O26^iv^	83.96 (15)
O30—V5—O32	87.86 (17)	O60—Na3—Na4	136.53 (13)
O30—V5—H31	81.4 (15)	O60—Na3—O31^iii^	90.25 (16)
O31—V5—V1	125.14 (15)	O60—Na3—O57	75.55 (17)
O31—V5—V10	112.38 (15)	O60—Na3—O26^iv^	104.97 (16)
O31—V5—V14	118.42 (15)	O31^iii^—Na3—Na4	117.15 (12)
O31—V5—O0P	108.49 (18)	O57—Na3—Na4	69.59 (13)
O31—V5—O36	110.26 (18)	O57—Na3—O31^iii^	94.51 (15)
O31—V5—O32	107.14 (19)	O57—Na3—O26^iv^	178.98 (17)
O31—V5—O30	108.20 (19)	O26^iv^—Na3—Na4	109.56 (12)
O31—V5—H31	33.2 (6)	O26^iv^—Na3—O31^iii^	86.37 (14)
V5—V1—V13	134.72 (4)	Na2^v^—Na9—H16^vi^	151 (2)
O0P—V1—V5	39.48 (10)	O40—Na9—Na2^v^	78.30 (13)
O0P—V1—V13	133.28 (11)	O40—Na9—O59	170.51 (19)
O0P—V1—O5	101.64 (15)	O40—Na9—O41	105.71 (17)
O0P—V1—O1	70.10 (14)	O40—Na9—O16^vi^	91.76 (17)
O36—V1—V5	39.94 (11)	O40—Na9—H16^vi^	112.2 (11)
O36—V1—V13	124.32 (11)	O40—Na9—O39	89.53 (18)
O36—V1—O0P	77.77 (15)	O40—Na9—O02J^v^	89.57 (17)
O36—V1—O5	157.13 (15)	O59—Na9—Na2^v^	109.79 (14)
O36—V1—O1	85.50 (14)	O59—Na9—O16^vi^	85.16 (16)
O5—V1—V5	130.64 (12)	O59—Na9—H16^vi^	63.2 (8)
O5—V1—V13	41.22 (11)	O59—Na9—O02J^v^	94.73 (17)
O5—V1—O1	73.09 (15)	O41—Na9—Na2^v^	56.54 (12)
O4—V1—V5	113.91 (14)	O41—Na9—O59	83.28 (17)
O4—V1—V13	111.23 (14)	O41—Na9—O16^vi^	89.62 (16)
O4—V1—O0P	100.14 (18)	O41—Na9—H16^vi^	94 (2)
O4—V1—O36	103.34 (17)	O41—Na9—O39	163.6 (2)
O4—V1—O5	99.28 (17)	O41—Na9—O02J^v^	82.43 (16)
O4—V1—O1	165.44 (17)	O16^vi^—Na9—Na2^v^	139.18 (13)
O4—V1—O23	103.35 (18)	O16^vi^—Na9—H16^vi^	23.2 (4)
O1—V1—V5	65.77 (10)	O16^vi^—Na9—O02J^v^	172.00 (17)
O1—V1—V13	71.50 (10)	O39—Na9—Na2^v^	123.01 (14)
O23—V1—V5	123.61 (11)	O39—Na9—O59	81.89 (18)
O23—V1—V13	38.61 (11)	O39—Na9—O16^vi^	96.02 (17)
O23—V1—O0P	156.11 (16)	O39—Na9—H16^vi^	85.4 (19)
O23—V1—O36	92.47 (15)	O39—Na9—O02J^v^	91.89 (17)
O23—V1—O5	78.95 (15)	O02J^v^—Na9—Na2^v^	33.74 (11)
O23—V1—O1	87.59 (15)	O02J^v^—Na9—H16^vi^	158.0 (8)
V6—V7—V4	74.22 (3)	Na1—Na2—Na9^vii^	86.54 (7)
O18—V7—V6	132.31 (13)	O015—Na2—Na1	80.23 (12)
O18—V7—V4	131.71 (13)	O015—Na2—Na9^vii^	138.09 (14)
O18—V7—O9	97.24 (17)	O01E—Na2—Na1	36.60 (11)
O18—V7—O19	94.86 (17)	O01E—Na2—Na9^vii^	68.54 (12)
O18—V7—O10	159.23 (17)	O01E—Na2—O015	77.59 (16)
O9—V7—V6	40.97 (11)	O01E—Na2—O01U	120.30 (18)
O9—V7—V4	114.65 (11)	O01E—Na2—O02J	76.54 (16)
O9—V7—O10	78.66 (15)	O01U—Na2—Na1	153.43 (15)
O19—V7—V6	110.33 (12)	O01U—Na2—Na9^vii^	95.13 (13)
O19—V7—V4	39.12 (11)	O01U—Na2—O015	80.96 (15)
O19—V7—O9	145.16 (16)	O01U—Na2—O02J	122.71 (18)
O19—V7—O10	78.53 (16)	O02J—Na2—Na1	71.99 (13)
O10—V7—V6	38.09 (11)	O02J—Na2—Na9^vii^	36.14 (12)
O10—V7—V4	39.46 (11)	O02J—Na2—O015	151.57 (18)
O015—V7—V6	109.19 (14)	O02K—Na2—Na1	90.62 (14)
O015—V7—V4	104.06 (14)	O02K—Na2—Na9^vii^	142.60 (15)
O015—V7—O18	101.83 (18)	O02K—Na2—O015	77.54 (16)
O015—V7—O9	104.16 (19)	O02K—Na2—O01E	124.56 (18)
O015—V7—O19	105.11 (18)	O02K—Na2—O01U	103.44 (18)
O015—V7—O10	98.91 (18)	O02K—Na2—O02J	108.13 (19)
V3—V8—V9	73.47 (3)	Na6—Na7—Na8	117.71 (9)
V3—V8—H16	98.8 (16)	O46—Na7—Na8	75.62 (13)
V9—V8—H16	85.0 (15)	O46—Na7—Na6	46.10 (12)
V15—V8—V3	73.98 (3)	O46—Na7—O43	82.81 (19)
V15—V8—V9	129.24 (4)	O46—Na7—O37	91.29 (17)
V15—V8—H16	138 (2)	O46—Na7—O39	121.39 (19)
O17—V8—V3	41.57 (11)	O46—Na7—O38	158.2 (2)
O17—V8—V9	114.85 (11)	O43—Na7—Na8	31.71 (13)
O17—V8—V15	42.65 (11)	O43—Na7—Na6	108.51 (16)
O17—V8—H16	105 (2)	O43—Na7—O37	145.1 (2)
O7—V8—V3	40.86 (11)	O43—Na7—O39	91.37 (19)
O7—V8—V9	38.00 (11)	O43—Na7—O38	93.7 (2)
O7—V8—V15	113.42 (11)	O37—Na7—Na8	166.56 (14)
O7—V8—O17	81.13 (16)	O37—Na7—Na6	48.95 (11)
O7—V8—H16	76.8 (7)	O39—Na7—Na8	70.35 (14)
O25—V8—V3	108.40 (12)	O39—Na7—Na6	152.22 (17)
O25—V8—V9	39.63 (11)	O39—Na7—O37	120.4 (2)
O25—V8—V15	124.30 (12)	O39—Na7—O38	80.05 (18)
O25—V8—O17	143.98 (16)	O38—Na7—Na8	111.94 (14)
O25—V8—O7	77.60 (16)	O38—Na7—Na6	116.55 (15)
O25—V8—H16	98 (2)	O38—Na7—O37	79.26 (17)
O15—V8—V3	110.93 (13)	V5—O0P—V1	98.70 (16)
O15—V8—V9	121.46 (12)	V5—O0P—V14	91.60 (16)
O15—V8—V15	43.06 (12)	V5—O0P—H0P	112.7
O15—V8—O17	85.70 (16)	V1—O0P—H0P	112.7
O15—V8—O7	138.76 (16)	V14—O0P—V1	124.71 (18)
O15—V8—O25	91.45 (17)	V14—O0P—H0P	112.7
O15—V8—H16	144.4 (7)	V5—O36—V1	99.21 (16)
O16—V8—V3	124.42 (16)	V5—O36—V10	101.80 (17)
O16—V8—V9	113.62 (14)	V5—O36—H36	106.5
O16—V8—V15	116.80 (14)	V1—O36—H36	106.5
O16—V8—O17	107.93 (19)	V10—O36—V1	133.55 (19)
O16—V8—O7	111.55 (18)	V10—O36—H36	106.5
O16—V8—O25	106.81 (19)	V3—O17—H17	111.6
O16—V8—O15	109.68 (19)	V8—O17—V3	97.82 (16)
O16—V8—H16	34.8 (6)	V8—O17—H17	111.6
V13—V9—V8	74.50 (3)	V15—O17—V3	126.31 (18)
O5—V9—V8	114.45 (12)	V15—O17—V8	94.41 (17)
O5—V9—V13	40.63 (11)	V15—O17—H17	111.6
O5—V9—O7	145.20 (16)	N1—O2—V2	128.8 (3)
O5—V9—O25	79.17 (16)	V7—O18—V15	127.2 (2)
O27—V9—V8	131.49 (13)	V7—O18—H18	109.0 (19)
O27—V9—V13	130.92 (13)	V15—O18—H18	121 (2)
O27—V9—O5	96.09 (17)	V2—O13—H13	112.4
O27—V9—O7	95.89 (17)	V11—O13—V2	124.04 (19)
O27—V9—O25	156.87 (17)	V11—O13—H13	112.4
O7—V9—V8	38.59 (11)	V12—O13—V2	97.72 (17)
O7—V9—V13	110.57 (12)	V12—O13—V11	94.50 (16)
O7—V9—O25	77.06 (16)	V12—O13—H13	112.4
O25—V9—V8	38.51 (11)	V1—O5—H5	111.6
O25—V9—V13	38.73 (11)	V9—O5—V1	122.9 (2)
O6—V9—V8	103.96 (14)	V9—O5—V13	100.59 (17)
O6—V9—V13	110.14 (15)	V9—O5—H5	111.6
O6—V9—O5	105.86 (19)	V13—O5—V1	96.33 (16)
O6—V9—O27	102.47 (19)	V13—O5—H5	111.6
O6—V9—O7	103.22 (18)	V6—O9—V3	96.13 (15)
O6—V9—O25	100.59 (18)	V6—O9—H9	112.5
V4—V10—V5	74.71 (3)	V3—O9—H9	112.5
O36—V10—V4	111.29 (12)	V7—O9—V6	99.86 (16)
O36—V10—V5	39.00 (11)	V7—O9—V3	121.09 (19)
O36—V10—O32	76.42 (15)	V7—O9—H9	112.5
O21—V10—V4	132.24 (13)	V2—O19—H19	104.8
O21—V10—V5	130.33 (13)	V4—O19—V2	100.14 (17)
O21—V10—O36	94.20 (17)	V4—O19—H19	104.8
O21—V10—O12	96.48 (17)	V7—O19—V2	136.3 (2)
O21—V10—O32	155.53 (17)	V7—O19—V4	102.51 (18)
O12—V10—V4	41.50 (12)	V7—O19—H19	104.8
O12—V10—V5	115.37 (12)	V9—O27—V14	128.0 (2)
O12—V10—O36	146.07 (16)	V9—O27—H27	109.3 (18)
O12—V10—O32	80.70 (16)	V14—O27—H27	122.0 (19)
O33—V10—V4	107.62 (15)	V6—O10—V4	142.6 (2)
O33—V10—V5	104.06 (15)	V6—O10—V7	101.09 (16)
O33—V10—O36	104.53 (17)	V6—O10—H10	102.6
O33—V10—O21	103.7 (2)	V4—O10—V7	99.85 (17)
O33—V10—O12	104.02 (18)	V4—O10—H10	102.6
O33—V10—O32	100.53 (19)	V7—O10—H10	102.6
O32—V10—V4	39.25 (11)	V6—O28—V3	102.54 (17)
O32—V10—V5	37.50 (11)	V6—O28—V14	94.30 (16)
V12—V11—V13	78.16 (3)	V6—O28—H28	104.1
O13—V11—V12	42.49 (11)	V3—O28—H28	104.1
O13—V11—V13	119.41 (12)	V14—O28—V3	142.4 (2)
O13—V11—O24	81.80 (16)	V14—O28—H28	104.1
O21—V11—V12	128.53 (12)	V10—O21—V11	128.3 (2)
O21—V11—V13	128.87 (12)	V10—O21—H21	108.8 (18)
O21—V11—O13	91.68 (16)	V11—O21—H21	122.8 (19)
O21—V11—O23	89.96 (16)	V2—O12—H12	111.6
O21—V11—O24	155.20 (18)	V4—O12—V2	97.34 (16)
O23—V11—V12	114.65 (12)	V4—O12—H12	111.6
O23—V11—V13	41.10 (11)	V10—O12—V2	123.49 (19)
O23—V11—O13	145.27 (17)	V10—O12—V4	98.83 (17)
O23—V11—O24	82.34 (15)	V10—O12—H12	111.6
O22—V11—V12	112.58 (15)	V3—O7—H7	105.5
O22—V11—V13	105.56 (15)	V8—O7—V3	98.72 (16)
O22—V11—O13	107.50 (18)	V8—O7—H7	105.5
O22—V11—O21	101.4 (2)	V9—O7—V3	135.08 (19)
O22—V11—O23	106.15 (19)	V9—O7—V8	103.40 (18)
O22—V11—O24	103.4 (2)	V9—O7—H7	105.5
O24—V11—V12	39.39 (11)	V2—O34—H34	106.1
O24—V11—V13	41.60 (11)	V12—O34—V2	100.76 (17)
V2—V12—H14	112 (2)	V12—O34—V15	97.69 (17)
V11—V12—V2	73.86 (3)	V12—O34—H34	106.1
V11—V12—V15	131.14 (4)	V15—O34—V2	136.5 (2)
V11—V12—H14	152.2 (11)	V15—O34—H34	106.1
V15—V12—V2	76.34 (3)	V7—O015—Na2	134.1 (2)
V15—V12—H14	75.6 (7)	Na4—O56—H56A	109.4
O13—V12—V2	42.15 (11)	Na4—O56—H56B	109.3
O13—V12—V11	43.00 (11)	H56A—O56—H56B	104.5
O13—V12—V15	118.40 (12)	Na5—O50—H50A	109.5
O13—V12—H14	123 (2)	Na5—O50—H50B	109.4
O34—V12—V2	40.36 (11)	H50A—O50—H50B	104.3
O34—V12—V11	113.18 (12)	N1—O3—V3	129.7 (3)
O34—V12—V15	41.75 (12)	N1—O1—V1	129.5 (4)
O34—V12—O13	80.66 (16)	Na1—O01E—Na2	106.18 (18)
O34—V12—H14	80.9 (15)	Na5—O52—Na4	84.44 (14)
O15—V12—V2	111.90 (13)	V8—O25—V9	101.86 (18)
O15—V12—V11	122.17 (12)	V8—O25—H25	101.3
O15—V12—V15	41.62 (12)	V9—O25—H25	101.3
O15—V12—O13	146.83 (17)	V13—O25—V8	144.0 (2)
O15—V12—O34	83.20 (17)	V13—O25—V9	100.73 (17)
O15—V12—H14	82.0 (19)	V13—O25—H25	101.3
O14—V12—V2	124.31 (15)	Na5—O53—Na4	84.43 (16)
O14—V12—V11	117.96 (15)	Na3—O55—Na4	121.95 (19)
O14—V12—V15	110.77 (15)	Na8—O45—Na1^ii^	82.10 (14)
O14—V12—O13	107.47 (18)	V1—O23—H23	104.0
O14—V12—O34	109.75 (18)	V11—O23—V1	140.7 (2)
O14—V12—O15	105.21 (19)	V11—O23—H23	104.0
O14—V12—H14	35.4 (6)	V13—O23—V1	102.02 (18)
O14—V12—O24	109.6 (2)	V13—O23—V11	97.45 (16)
O24—V12—V2	111.35 (13)	V13—O23—H23	104.0
O24—V12—V11	43.03 (12)	Na5—O51—Na4	83.94 (14)
O24—V12—V15	121.91 (14)	Na6—O48—H48A	109.5
O24—V12—O13	85.94 (17)	Na6—O48—H48B	109.4
O24—V12—O34	140.58 (17)	H48A—O48—H48B	104.5
O24—V12—O15	88.45 (17)	Na3—O58—H58A	109.5
O24—V12—H14	136 (2)	Na3—O58—H58B	109.3
V1—V13—V9	70.66 (3)	H58A—O58—H58B	104.5
V1—V13—H26	157.6 (9)	Na1—O01P—Na8^i^	80.03 (14)
V9—V13—H26	111 (2)	V4—O32—V10	99.99 (17)
V11—V13—V1	76.55 (3)	V4—O32—H32	99.8
V11—V13—V9	128.88 (4)	V5—O32—V4	146.2 (2)
V11—V13—H26	114 (2)	V5—O32—V10	103.57 (18)
O5—V13—V1	42.45 (11)	V5—O32—H32	99.8
O5—V13—V9	38.77 (11)	V10—O32—H32	99.8
O5—V13—V11	118.67 (11)	Na4—O54—H54A	109.7
O5—V13—H26	126 (2)	Na4—O54—H54B	109.5
O25—V13—V1	106.68 (12)	H54A—O54—H54B	104.4
O25—V13—V9	40.54 (11)	Na9—O40—H40A	109.4
O25—V13—V11	123.37 (12)	Na9—O40—H40B	109.6
O25—V13—O5	79.13 (16)	H40A—O40—H40B	104.5
O25—V13—O23	135.44 (17)	Na2—O01U—H01A	109.7
O25—V13—O24	90.83 (17)	Na2—O01U—H01B	109.4
O25—V13—H26	84.8 (17)	H01A—O01U—H01B	104.5
O23—V13—V1	39.37 (12)	V6—O30—V14	92.76 (15)
O23—V13—V9	108.39 (12)	V6—O30—H30	103.0
O23—V13—V11	41.46 (11)	V5—O30—V6	151.6 (2)
O23—V13—O5	80.91 (16)	V5—O30—V14	92.58 (16)
O23—V13—O24	84.25 (16)	V5—O30—H30	103.0
O23—V13—H26	138 (2)	V14—O30—H30	103.0
O24—V13—V1	113.51 (12)	H01C—O01W—H01D	104.6
O24—V13—V9	121.99 (13)	Na8—O44—H44A	109.8
O24—V13—V11	43.17 (12)	Na8—O44—H44B	109.8
O24—V13—O5	146.22 (17)	H44A—O44—H44B	104.1
O24—V13—H26	84.8 (18)	Na8—O59—Na9	125.75 (19)
O26—V13—V1	123.38 (14)	Na6—O49—Na5	123.3 (2)
O26—V13—V9	116.66 (15)	Na3—O60—H60A	109.3
O26—V13—V11	113.99 (15)	Na3—O60—H60B	109.5
O26—V13—O5	106.19 (18)	H60A—O60—H60B	104.3
O26—V13—O25	110.00 (19)	Na1—O022—H02A	109.6
O26—V13—O23	113.70 (19)	Na1—O022—H02B	109.7
O26—V13—O24	107.55 (19)	H02A—O022—H02B	104.3
O26—V13—H26	34.7 (6)	Na1—O023—Na8^i^	82.71 (15)
V5—V14—V6	80.94 (3)	Na6—O47—H47A	109.9
O0P—V14—V6	123.24 (11)	Na6—O47—H47B	109.6
O0P—V14—V5	43.82 (11)	H47A—O47—H47B	104.1
O0P—V14—O30	85.64 (15)	Na1—O025—H02C	109.4
O27—V14—V6	128.10 (12)	Na1—O025—H02D	109.4
O27—V14—V5	128.61 (13)	H02C—O025—H02D	104.5
O27—V14—O0P	89.85 (16)	V8—O15—V15	95.83 (18)
O27—V14—O28	87.68 (16)	V8—O15—H15	101.5
O27—V14—O30	156.89 (16)	V12—O15—V8	149.3 (2)
O28—V14—V6	42.11 (11)	V12—O15—V15	99.18 (18)
O28—V14—V5	117.87 (11)	V12—O15—H15	101.5
O28—V14—O0P	146.70 (16)	V15—O15—H15	101.5
O28—V14—O30	83.81 (15)	Na7—O46—Na6	89.32 (17)
O29—V14—V6	104.86 (15)	V12—O14—Na6^i^	128.8 (2)
O29—V14—V5	111.83 (14)	V12—O14—H14	74.0 (17)
O29—V14—O0P	106.96 (18)	Na6^i^—O14—H14	71 (3)
O29—V14—O27	100.78 (18)	Na7—O43—Na8	117.8 (2)
O29—V14—O28	106.12 (19)	Na9—O41—H41A	109.4
O29—V14—O30	102.22 (18)	Na9—O41—H41B	109.3
O30—V14—V6	42.06 (11)	H41A—O41—H41B	104.5
O30—V14—V5	41.88 (11)	V11—O24—H24	102.1
V8—V15—V12	77.68 (3)	V12—O24—V11	97.58 (17)
O17—V15—V8	42.94 (11)	V12—O24—V13	149.5 (2)
O17—V15—V12	119.02 (12)	V12—O24—H24	102.1
O17—V15—O34	145.80 (16)	V13—O24—V11	95.23 (17)
O17—V15—O15	84.04 (16)	V13—O24—H24	102.1
O18—V15—V8	128.64 (12)	V8—O16—Na9^viii^	134.1 (2)
O18—V15—V12	128.95 (12)	V8—O16—H16	79.3 (17)
O18—V15—O17	90.93 (16)	Na9^viii^—O16—H16	54.9 (17)
O18—V15—O34	91.22 (16)	V5—O31—Na3^ix^	140.5 (2)
O18—V15—O15	154.65 (16)	V5—O31—H31	77.9 (18)
O34—V15—V8	114.65 (11)	Na3^ix^—O31—H31	82 (3)
O34—V15—V12	40.57 (11)	V6—O37—Na6	134.6 (2)
O34—V15—O15	79.61 (16)	V6—O37—Na7	138.4 (2)
O15—V15—V8	41.11 (12)	Na7—O37—Na6	85.08 (15)
O15—V15—V12	39.20 (11)	Na5—O02G—H02E	109.4
O35—V15—V8	111.79 (14)	Na5—O02G—H02F	109.6
O35—V15—V12	106.96 (15)	H02E—O02G—H02F	104.3
O35—V15—O17	107.04 (18)	Na7—O39—Na9	135.0 (2)
O35—V15—O18	101.20 (18)	Na7—O38—H38A	109.6
O35—V15—O34	105.99 (18)	Na7—O38—H38B	109.5
O35—V15—O15	104.03 (18)	H38A—O38—H38B	104.4
Na8^i^—Na1—Na2	138.12 (9)	Na2—O02J—Na9^vii^	110.12 (19)
O01E—Na1—Na8^i^	116.28 (14)	Na2—O02K—H02G	109.9
O01E—Na1—Na2	37.22 (12)	Na2—O02K—H02H	109.6
O01E—Na1—O45^i^	91.45 (15)	H02G—O02K—H02H	103.9
O01E—Na1—O01P	167.73 (18)	Na3—O57—H57A	109.3
O01E—Na1—O022	87.47 (16)	Na3—O57—H57B	109.3
O01E—Na1—O023	85.63 (16)	H57A—O57—H57B	104.5
O45^i^—Na1—Na8^i^	48.15 (11)	V13—O26—Na3^x^	135.9 (2)
O45^i^—Na1—Na2	93.16 (12)	V13—O26—H26	81.8 (17)
O01P—Na1—Na8^i^	51.68 (11)	Na3^x^—O26—H26	130 (3)
O01P—Na1—Na2	152.34 (14)	O3—N1—O2	120.0 (4)
O01P—Na1—O45^i^	81.31 (15)	O3—N1—O1	120.4 (5)
O01P—Na1—O022	99.45 (16)	O1—N1—O2	119.6 (5)
			
V2—V4—O32—V5	102.7 (4)	O13—V12—O24—V11	−3.22 (18)
V2—V4—O32—V10	−31.00 (17)	O13—V12—O24—V13	−117.2 (5)
V2—V12—O15—V8	84.9 (5)	O5—V9—O27—V14	−78.0 (3)
V2—V12—O15—V15	−33.33 (17)	O5—V13—O26—Na3^x^	12.0 (4)
V2—V12—O14—Na6^i^	128.6 (2)	O9—V6—O10—V4	129.7 (4)
V2—V12—O24—V11	31.11 (19)	O9—V6—O10—V7	6.78 (16)
V2—V12—O24—V13	−82.9 (5)	O9—V6—O28—V3	−11.58 (17)
V2—O2—N1—O3	−8.6 (8)	O9—V6—O28—V14	−157.70 (18)
V2—O2—N1—O1	171.2 (3)	O9—V6—O30—V5	−24.9 (6)
V6—V7—O18—V15	53.9 (3)	O9—V6—O30—V14	75.7 (3)
V6—V7—O015—Na2	−83.2 (3)	O9—V6—O37—Na6	−5.6 (3)
V4—V7—O18—V15	−54.7 (3)	O9—V6—O37—Na7	−164.1 (3)
V4—V7—O015—Na2	−5.3 (3)	O9—V7—O18—V15	77.8 (3)
V4—V10—O21—V11	52.9 (3)	O9—V7—O015—Na2	−125.7 (3)
V3—V6—O10—V4	97.1 (3)	O19—V4—O32—V5	69.4 (5)
V3—V6—O10—V7	−25.82 (17)	O19—V4—O32—V10	−64.3 (3)
V3—V6—O28—V14	−146.1 (3)	O19—V7—O18—V15	−69.4 (3)
V3—V6—O30—V5	−66.0 (5)	O19—V7—O015—Na2	35.2 (3)
V3—V6—O30—V14	34.57 (17)	O10—V6—O28—V3	52.5 (3)
V3—V6—O37—Na6	37.0 (3)	O10—V6—O28—V14	−93.6 (2)
V3—V6—O37—Na7	−121.5 (3)	O10—V6—O30—V5	42.7 (4)
V3—V8—O15—V12	−86.4 (5)	O10—V6—O30—V14	143.28 (16)
V3—V8—O15—V15	32.64 (16)	O10—V6—O37—Na6	−89.8 (3)
V3—V8—O16—Na9^viii^	44.9 (4)	O10—V6—O37—Na7	111.7 (3)
V3—O3—N1—O2	−177.2 (3)	O10—V4—O32—V5	−9.4 (4)
V3—O3—N1—O1	3.1 (8)	O10—V4—O32—V10	−143.12 (17)
V5—V10—O21—V11	−54.8 (3)	O10—V7—O18—V15	0.8 (7)
V1—V5—O32—V4	−100.9 (4)	O10—V7—O015—Na2	−45.2 (3)
V1—V5—O32—V10	32.02 (18)	O28—V6—O10—V4	65.1 (4)
V1—V5—O30—V6	68.3 (5)	O28—V6—O10—V7	−57.8 (3)
V1—V5—O30—V14	−32.31 (16)	O28—V6—O30—V5	−94.0 (4)
V1—V5—O31—Na3^ix^	−121.1 (3)	O28—V6—O30—V14	6.62 (17)
V1—V13—O26—Na3^x^	−31.2 (4)	O28—V6—O37—Na6	80.1 (3)
V1—O1—N1—O2	7.2 (8)	O28—V6—O37—Na7	−78.3 (4)
V1—O1—N1—O3	−173.0 (3)	O12—V4—O32—V5	136.0 (4)
V7—V6—O10—V4	122.9 (4)	O12—V4—O32—V10	2.28 (16)
V7—V6—O28—V3	16.95 (18)	O12—V10—O21—V11	76.7 (3)
V7—V6—O28—V14	−129.17 (11)	O7—V8—O15—V12	−48.8 (6)
V7—V6—O30—V5	16.2 (5)	O7—V8—O15—V15	70.3 (3)
V7—V6—O30—V14	116.82 (11)	O7—V8—O16—Na9^viii^	0.5 (4)
V7—V6—O37—Na6	−45.5 (3)	O7—V9—O27—V14	69.3 (3)
V7—V6—O37—Na7	156.0 (3)	O34—V12—O15—V8	113.9 (5)
V7—V4—O32—V5	22.0 (5)	O34—V12—O15—V15	−4.37 (16)
V7—V4—O32—V10	−111.74 (14)	O34—V12—O14—Na6^i^	85.9 (3)
V8—V9—O27—V14	52.6 (3)	O34—V12—O24—V11	66.7 (3)
V8—V15—O18—V7	−53.5 (3)	O34—V12—O24—V13	−47.2 (6)
V9—V8—O15—V12	−3.5 (5)	O34—V15—O18—V7	70.0 (3)
V9—V8—O15—V15	115.56 (12)	O015—V7—O18—V15	−176.0 (3)
V9—V8—O16—Na9^viii^	−40.7 (3)	O25—V8—O15—V12	24.0 (5)
V9—V13—O26—Na3^x^	52.3 (3)	O25—V8—O15—V15	142.99 (16)
V10—V4—O32—V5	133.7 (5)	O25—V8—O16—Na9^viii^	−82.5 (3)
V10—V5—O32—V4	−132.9 (5)	O25—V9—O27—V14	−1.4 (6)
V10—V5—O30—V6	−16.5 (5)	O25—V13—O26—Na3^x^	96.1 (3)
V10—V5—O30—V14	−117.12 (11)	O23—V13—O26—Na3^x^	−75.0 (3)
V10—V5—O31—Na3^ix^	−35.5 (4)	O33—V10—O21—V11	−177.1 (2)
V11—V12—O15—V8	0.7 (5)	O32—V5—O30—V6	−39.7 (4)
V11—V12—O15—V15	−117.53 (12)	O32—V5—O30—V14	−140.37 (17)
V11—V12—O14—Na6^i^	−142.50 (19)	O32—V5—O31—Na3^ix^	5.6 (4)
V11—V12—O24—V13	−114.0 (5)	O32—V10—O21—V11	−5.1 (6)
V11—V13—O26—Na3^x^	−120.5 (3)	O30—V6—O10—V4	−21.4 (3)
V12—V15—O18—V7	53.7 (3)	O30—V6—O10—V7	−144.32 (18)
V13—V9—O27—V14	−54.6 (3)	O30—V6—O28—V3	139.31 (19)
V14—V6—O10—V4	9.3 (4)	O30—V6—O28—V14	−6.81 (17)
V14—V6—O10—V7	−113.65 (13)	O30—V6—O37—Na6	174.9 (3)
V14—V6—O28—V3	146.1 (3)	O30—V6—O37—Na7	16.5 (4)
V14—V6—O30—V5	−100.6 (5)	O30—V5—O32—V4	14.1 (4)
V14—V6—O37—Na6	127.3 (2)	O30—V5—O32—V10	146.99 (19)
V14—V6—O37—Na7	−31.2 (4)	O30—V5—O31—Na3^ix^	99.0 (3)
V14—V5—O32—V4	−18.2 (5)	O6—V9—O27—V14	174.3 (3)
V14—V5—O32—V10	114.70 (14)	O15—V8—O16—Na9^viii^	179.7 (3)
V14—V5—O30—V6	100.6 (5)	O15—V12—O14—Na6^i^	−2.2 (3)
V14—V5—O31—Na3^ix^	147.7 (3)	O15—V12—O24—V11	144.06 (19)
V15—V8—O15—V12	−119.0 (5)	O15—V12—O24—V13	30.1 (5)
V15—V8—O16—Na9^viii^	133.2 (2)	O15—V15—O18—V7	2.1 (6)
V15—V12—O15—V8	118.2 (5)	O14—V12—O15—V8	−137.5 (4)
V15—V12—O14—Na6^i^	41.3 (3)	O14—V12—O15—V15	104.3 (2)
V15—V12—O24—V11	117.94 (13)	O14—V12—O24—V11	−110.3 (2)
V15—V12—O24—V13	4.0 (5)	O14—V12—O24—V13	135.7 (4)
O0P—V5—O32—V4	−73.2 (5)	O24—V12—O15—V8	−27.5 (5)
O0P—V5—O32—V10	59.8 (3)	O24—V12—O15—V15	−145.76 (18)
O0P—V5—O30—V6	103.4 (4)	O24—V12—O14—Na6^i^	−96.1 (3)
O0P—V5—O30—V14	2.77 (16)	O24—V13—O26—Na3^x^	−166.3 (3)
O0P—V5—O31—Na3^ix^	−164.6 (3)	O16—V8—O15—V12	132.4 (4)
O36—V5—O32—V4	−129.8 (4)	O16—V8—O15—V15	−108.6 (2)
O36—V5—O32—V10	3.15 (17)	O35—V15—O18—V7	176.5 (3)
O36—V5—O30—V6	28.0 (6)	O31—V5—O32—V4	122.5 (4)
O36—V5—O30—V14	−72.6 (3)	O31—V5—O32—V10	−104.6 (2)
O36—V5—O31—Na3^ix^	−77.7 (4)	O31—V5—O30—V6	−147.1 (4)
O36—V10—O21—V11	−71.1 (3)	O31—V5—O30—V14	112.29 (18)
O17—V8—O15—V12	−120.1 (5)	O37—V6—O10—V4	−128.5 (3)
O17—V8—O15—V15	−1.06 (16)	O37—V6—O10—V7	108.58 (19)
O17—V8—O16—Na9^viii^	87.8 (3)	O37—V6—O28—V3	−113.8 (2)
O17—V15—O18—V7	−75.9 (3)	O37—V6—O28—V14	100.1 (2)
O18—V7—O015—Na2	133.6 (3)	O37—V6—O30—V5	154.0 (4)
O13—V12—O15—V8	52.6 (6)	O37—V6—O30—V14	−105.38 (19)
O13—V12—O15—V15	−65.6 (3)	O11—V4—O32—V5	−117.4 (4)
O13—V12—O14—Na6^i^	172.0 (2)	O11—V4—O32—V10	108.9 (2)

Symmetry codes: (i) *x*−1/2, −*y*+3/2, *z*−1/2; (ii) *x*+1/2, −*y*+3/2, *z*+1/2; (iii) *x*+1/2, −*y*+3/2, *z*−1/2; (iv) *x*+1/2, *y*+1/2, *z*; (v) *x*+1/2, *y*−1/2, *z*; (vi) *x*, −*y*+1, *z*+1/2; (vii) *x*−1/2, *y*+1/2, *z*; (viii) *x*, −*y*+1, *z*−1/2; (ix) *x*−1/2, −*y*+3/2, *z*+1/2; (x) *x*−1/2, *y*−1/2, *z*.

### Poly[[heptadecaaquatetradecaoxidononasodium] [pentacosaaquanitratoundecaoxidopentadecavanadium]] Hydrogen-bond geometry (Å, º)

**Table:**

*D*—H···*A*	*D*—H	H···*A*	*D*···*A*	*D*—H···*A*
O0*P*—H0*P*···O02*G*^x^	1.00	1.76	2.752 (5)	174
O36—H36···O025^x^	1.00	1.80	2.803 (5)	177
O17—H17···O56	1.00	1.74	2.726 (5)	169
O13—H13···O54^xi^	1.00	1.74	2.715 (6)	165
O5—H5···O55^x^	1.00	1.87	2.791 (5)	152
O9—H9···O49	1.00	1.80	2.729 (6)	153
O19—H19···O01*E*	1.00	1.82	2.789 (5)	163
O27—H27···O023^v^	0.87 (1)	2.07 (5)	2.790 (6)	139 (6)
O10—H10···O01*U*	1.00	1.86	2.857 (6)	174
O12—H12···O50^xi^	1.00	1.73	2.718 (5)	169
O7—H7···O59^viii^	1.00	1.76	2.751 (6)	173
O56—H56*A*···O17	0.87	1.88	2.726 (5)	165
O56—H56*B*···O57	0.87	1.97	2.753 (6)	148
O50—H50*A*···O12^xii^	0.87	1.91	2.718 (5)	154
O50—H50*B*···O47	0.87	1.91	2.766 (6)	168
O48—H48*A*···O33^xii^	0.87	1.85	2.719 (6)	174
O58—H58*B*···O35	0.87	2.01	2.748 (6)	142
O54—H54*A*···O13^xii^	0.87	2.01	2.715 (6)	137
O54—H54*B*···O38^iii^	0.87	1.93	2.745 (6)	155
O40—H40*A*···O50^x^	0.87	1.89	2.727 (6)	160
O40—H40*B*···O01*E*^v^	0.87	1.93	2.752 (6)	156
O01*U*—H01*A*···O10	0.87	2.04	2.857 (6)	157
O01*W*—H01*C*···O02*G*	0.87	2.06	2.795 (6)	142
O01*W*—H01*D*···O31^iv^	0.87	2.05	2.883 (6)	161
O44—H44*B*···O58^ii^	0.88	1.88	2.740 (6)	168
O60—H60*A*···O46^i^	0.87	1.92	2.766 (6)	164
O60—H60*B*···O33^iii^	0.87	2.00	2.860 (6)	167
O022—H02*A*···O48^vii^	0.88	1.98	2.795 (6)	154
O022—H02*B*···O02*J*	0.87	2.09	2.895 (6)	152
O47—H47*A*···O50	0.88	2.05	2.766 (6)	139
O47—H47*B*···O57^ii^	0.88	2.14	2.864 (6)	139
O025—H02*C*···O60^xiii^	0.87	1.90	2.763 (6)	169
O025—H02*D*···O02*K*	0.87	1.95	2.791 (7)	162
O41—H41*A*···O11^v^	0.87	1.91	2.746 (6)	159
O41—H41*B*···O56^vi^	0.87	1.92	2.760 (6)	162
O02*G*—H02*E*···O01*W*	0.87	2.05	2.795 (6)	142
O02*G*—H02*F*···O02*K*	0.87	2.02	2.828 (7)	154
O38—H38*A*···O54^ix^	0.87	1.88	2.745 (6)	175
O38—H38*B*···O55^ix^	0.87	2.22	2.832 (6)	127
O02*K*—H02*G*···O26^ii^	0.88	2.21	2.769 (6)	121
O57—H57*A*···O56	0.87	1.89	2.753 (6)	171
O57—H57*B*···O47^i^	0.87	2.10	2.864 (6)	146

Symmetry codes: (i) *x*−1/2, −*y*+3/2, *z*−1/2; (ii) *x*+1/2, −*y*+3/2, *z*+1/2; (iii) *x*+1/2, −*y*+3/2, *z*−1/2; (iv) *x*+1/2, *y*+1/2, *z*; (v) *x*+1/2, *y*−1/2, *z*; (vi) *x*, −*y*+1, *z*+1/2; (vii) *x*−1/2, *y*+1/2, *z*; (viii) *x*, −*y*+1, *z*−1/2; (ix) *x*−1/2, −*y*+3/2, *z*+1/2; (x) *x*−1/2, *y*−1/2, *z*; (xi) *x*−1, *y*, *z*; (xii) *x*+1, *y*, *z*; (xiii) *x*, −*y*+2, *z*+1/2.
